# An Investigation of the Molecular Mechanisms Underlying the Analgesic Effect of Jakyak-Gamcho Decoction: A Network Pharmacology Study

**DOI:** 10.1155/2020/6628641

**Published:** 2020-12-01

**Authors:** Ho-Sung Lee, In-Hee Lee, Kyungrae Kang, Sang-In Park, Tae-Wook Kwon, Dae-Yeon Lee

**Affiliations:** ^1^The Fore, 87 Ogeum-ro, Songpa-gu, Seoul 05542, Republic of Korea; ^2^Forest Hospital, 129 Ogeum-ro, Songpa-gu, Seoul 05549, Republic of Korea; ^3^Forestheal Hospital, 173 Ogeum-ro, Songpa-gu, Seoul 05641, Republic of Korea

## Abstract

Herbal drugs have drawn substantial interest as effective analgesic agents; however, their therapeutic mechanisms remain to be fully understood. To address this question, we performed a network pharmacology study to explore the system-level mechanisms that underlie the analgesic activity of Jakyak-Gamcho decoction (JGd; Shaoyao-Gancao-Tang in Chinese and Shakuyaku-Kanzo-To in Japanese), an herbal prescription consisting of *Paeonia lactiflora* Pallas and *Glycyrrhiza uralensis* Fischer. Based on comprehensive information regarding the pharmacological and chemical properties of the herbal constituents of JGd, we identified 57 active chemical compounds and their 70 pain-associated targets. The JGd targets were determined to be involved in the regulation of diverse biological activities as follows: calcium- and cytokine-mediated signalings, calcium ion concentration and homeostasis, cellular behaviors of muscle and neuronal cells, inflammatory response, and response to chemical, cytokine, drug, and oxidative stress. The targets were further enriched in various pain-associated signalings, including the PI3K-Akt, estrogen, ErbB, neurotrophin, neuroactive ligand-receptor interaction, HIF-1, serotonergic synapse, JAK-STAT, and cAMP pathways. Thus, these data provide a systematic basis to understand the molecular mechanisms underlying the analgesic activity of herbal drugs.

## 1. Introduction

Pain is a major healthcare and socioeconomic issue worldwide that severely affects the overall health, quality of life, daily activities, and productivity of patients, and it places a substantial financial burden on healthcare systems and society [1–6]. Based on the pathophysiological mechanisms, pain is classified into (i) nociceptive and (ii) nonnociceptive neuropathic pain [7–21]. Nociceptive pain is caused by the activation and stimulation of nociceptors and pain pathways driven by inflammation, chemicals, or physical events, and it is subdivided into somatic and visceral [7–21]. Neuropathic pain develops due to damage, injury, dysfunction, or disease of the somatosensory nervous system, and it is further classified into central and peripheral [7–21]. At present, opioid analgesics, nonsteroidal anti-inflammatory drugs (NSAIDs), and non-anti-inflammatory antipyretic analgesic agents serve as primary therapies for pain alleviation [6, 15, 22–28]. However, current treatment options for pain management are still associated with limited efficacy and unwanted adverse effects [6, 15, 22–28]. Meanwhile, herbal drugs and multicomponent-multitarget-multipathway polypharmacological therapeutics have received considerable attention for pain treatment because of their important analgesic effects with fewer side effects and toxicity [29–36].

Jakyak-Gamcho decoction (JGd; Shaoyao-Gancao-Tang in Chinese and Shakuyaku-Kanzo-To in Japanese) is an herbal drug that consists of *Paeonia lactiflora* Pallas and *Glycyrrhiza uralensis* Fischer, which has been prescribed for the treatment of various types of pain, gynecological diseases, arthritic diseases (e.g., osteoarthritis and arthralgia), and muscular diseases (myalgia, muscle tension, spasm, and cramps) [30, 37–48]. Previous studies have demonstrated the various therapeutic properties of JGd, including its analgesic, anti-inflammatory, antispasmodic, and antiallergic effects [30, 37–40, 43, 44, 46–55]. Among the diverse effects of this herbal drug, the most common therapeutic use of JGd is to alleviate pain arising from cancer, diabetes, neuropathy, and muscle and arthritis diseases [30, 37–40, 44, 46–48], which makes it one of the most frequently prescribed oral analgesic agents in East Asia [56]. The analgesic mechanisms of JGd include the modulation of spinal *α*2-adrenoceptors, transient receptor potential vanilloid 1 (TRPV1) channels, calcium and Sirt1 signalings, muscle contraction and relaxation, and chemokine and cytokine expression [39, 44, 46, 51, 57–60]. However, the pharmacological properties of JGd at the systemic level need to be explored.

Because of the complex pharmacological nature of multicompound-multitarget-multipathway agents, there is often a fundamental limitation in investigating their comprehensive mechanisms of action based only on conventional biological experimental methodologies [61–68]. To overcome such challenges, network pharmacology, an integrative research field that systematically combines computational systems biology, network science, medicine, pharmacology, mathematics, and physics, has emerged as one of the most effective approaches for the mechanistic exploration of polypharmacological drugs, such as herbal medicines [61–68]. The goal of this integrative science is to unveil the mechanisms of disease pathogenesis and drug activity that are coordinated through the interactions among diverse biological components such as genes, proteins, cells, tissues, and organs [61–68]. Previous network pharmacology studies successfully investigated the polypharmacological properties of herbal drugs by identifying their active compounds and key therapeutic targets and further elucidating the distinct system-level pharmacological effects and mechanisms (e.g., therapeutic modulation of biological processes such as proliferation, apoptosis, cell cycle regulation, angiogenesis, oxidation and reduction, insulin metabolism, and inflammation) for the treatment of various diseases, including cancer, diabetes, arthritis, and ischemic stroke, which are exerted by the synergistic interplay between multiple compounds and targets contained in herbal drugs [61–79]. In the present network pharmacology study, we aimed to uncover the molecular mechanisms that underlie the analgesic properties of JGd with a system's perspective.

## 2. Materials and Methods

### 2.1. Screening of Active Chemical Compounds in Jakyak-Gamcho Decoction

Information on chemical compounds comprising the herbal constituents of JGd was investigated using the Traditional Chinese Medicine Systems Pharmacology (TCMSP) database [80]. Then, based on their absorption, distribution, metabolism, and excretion (ADME) properties (i.e., oral bioavailability (OB), Caco-2 permeability, and drug-likeness (DL)), chemical compounds that satisfy the following criteria were screened and determined to be bioactive as previously suggested [63, 80, 81] using the TCMSP [80]: OB ≥ 30%, Caco-2 permeability ≥ -0.4, and DL ≥ 0.18. In brief, OB is the proportion of orally administered drug compounds that enter the general circulation, and it is one of the most crucial considerations in the design and development of a drug [80, 82]. Of note, compounds with an OB larger than 30% are commonly regarded as effectively absorbed in the human body [80, 82]. Caco-2 permeability is an important index for the investigation of intestinal permeability and drug efflux that is based on an evaluation of the rate of absorption and diffusion of a compound across Caco-2 human intestinal cells [80, 83–85]. In general, a chemical compound is considered not permeable in the intestinal epithelium if its Caco-2 permeability is lower than −0.4 [86, 87]. DL is a widely used measurement that qualitatively assesses whether a certain compound is physicochemically and structurally suitable for use as a drug [80, 88]. Note that the average DL of all drugs is 0.18, and therefore, it is commonly used as the threshold to determine the pharmacological potential of a compound [80, 88].

### 2.2. Target Identification

Human targets of the active chemical compounds of JGd were investigated using various databases and models, including the PharmMapper [89], search tool for interactions of chemicals (STITCH) 5 [90], Swiss Target Prediction [91], similarity ensemble approach (SEA) [92], systematic drug targeting tool (SysDT) [93], and weighted ensemble similarity (WES) [94]. The pain-associated human genes and proteins were investigated from the DisGeNET [95], Therapeutic Target Database [96], GeneCards [97], Comparative Toxicogenomics Database [98], Human Genome Epidemiology Navigator [99], Online Mendelian Inheritance in Man [100], Pharmacogenomics Knowledgebase [101], and DrugBank [102], using the medical subject headings term “Pain” (ID: D010146) for *Homo sapiens* species.

### 2.3. Network Construction

The herbal medicine-active chemical compound (H-C), active chemical compound-target (C-T), and target-pathway (T-P) networks were generated by connecting the herbal medicines with their active chemical compounds, the compounds with their targets, and the targets with the signaling pathways in which they are enriched. The protein-protein interaction (PPI) network was generated using the STRING database (interaction confidence score ≥ 0.9) [103]. Analysis and visualization of networks were performed with Cytoscape software [104]. A network is composed of nodes (e.g., herbal medicines, chemical compounds, targets, or pathways) and edges (or links) describing the interactions among the nodes [105]. The degree is defined as the number of links of a node [105].

### 2.4. Contribution Index Evaluation

The network-based efficacy-based contribution index (CI) of active chemical compounds of JGd was evaluated following previous procedures as follows [81]:

(1) $$\begin{array}{c} \text{NE}\left(j\right)=\sum_{i=1}^{n}d_{i},\\ \text{CI}\left(j\right)=\frac{c_{j}\times \text{NE}\left(j\right)}{\sum_{i=1}^{m}c_{i}\times \text{NE}\left(i\right)}\times 100\%, \end{array}$$

where *m* is the number of chemical compounds, *n* is the number of targets of chemical compound *j*, *d*_*i*_ is the number of links of target *i* of chemical compound *j*, and *c*_*i*_ (or *c*_*j*_) is the number of previous studies having “pain” and component *i* (or *j*) in their title or abstract searched from the PubMed database (https://pubmed.ncbi.nlm.nih.gov/). The chemical compounds with the highest CIs were regarded as contributing more to the pharmacological activity of a certain herbal drug [81].

### 2.5. Functional Enrichment Analysis

Gene ontology (GO) enrichment analysis was performed with g:Profiler [106]. Pathway enrichment analysis was performed with Kyoto Encyclopedia of Genes and Genomes database [107]. Functional association analysis was conducted using GeneMANIA [108].

### 2.6. Molecular Docking Analysis

The structures of chemical compounds of JGd and their targets were obtained from the PubChem [109] and RCSB Protein Databank [110] databases, respectively. Then, the molecular docking scores between the chemical compounds and the targets were assessed using AutoDock Vina [111]. Of note, a certain chemical compound is regarded as having high binding affinity to a target if the corresponding docking score is less than or equal to −5.0 [112, 113].

## 3. Results

**Figure 1:**
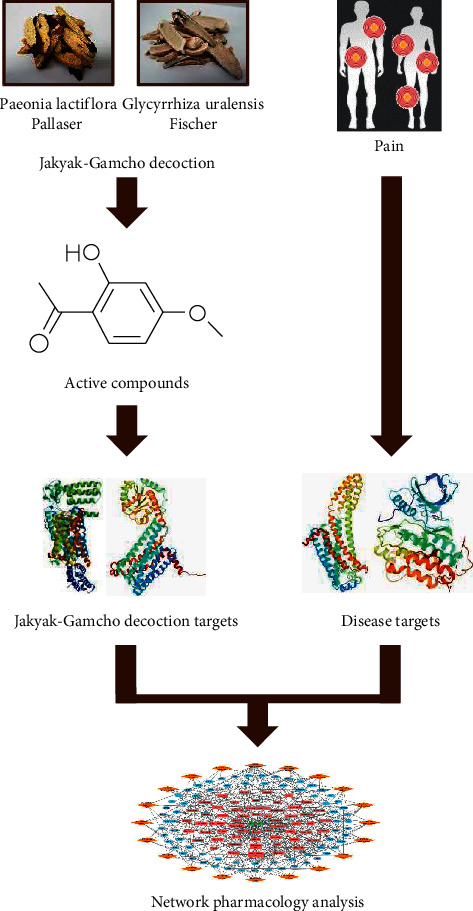
A schematic diagram illustrating the network pharmacology exploration of the analgesic mechanisms of Jakyak-Gamcho decoction.

The network pharmacology study for the exploration of analgesic mechanisms of JGd was conducted as follows (Figure 1). Detailed information regarding the chemical constituents of JGd was obtained from the comprehensive biomolecular databases, and the bioactive compounds were investigated using their ADME characteristics (Figure 1). The human targets of the active chemical compounds were identified from various databases and models that assess chemical-protein interactions (Figure 1). Then, we integrated the extensive herbal drug-related data into networks and performed network pharmacology analysis (Figure 1).

### 3.1. Active Chemical Compounds of Jakyak-Gamcho Decoction

Detailed information regarding the chemical compounds present in JGd was obtained from TCMSP [80] (Supplementary Table S1), and the active compounds were defined as those with OB ≥ 30%, Caco-2 permeability ≥ −0.4, and DL ≥ 0.18, as described previously [63, 80, 81]. Some components were also determined to be active because of the substantial amount contained in JGd and their reported relevant pharmacological activity [42, 57, 114–128], although they did not meet the criteria. As a result, 111 active chemical compounds were obtained for JGd (Supplementary Table S2).

### 3.2. Targets of Jakyak-Gamcho Decoction

We identified the targets of the active chemical compounds of JGd using the following databases and models for the investigation of chemical-protein interactions: Swiss Target Prediction [91], STITCH 5 [90], PharmMapper [89], SEA [92], SysDT [93], and WES [94]. Therefore, 70 human pain-associated and 137 nonpain-associated targets were obtained for JGd (Supplementary Table S3).

### 3.3. Network Pharmacology-Based Analysis of Jakyak-Gamcho Decoction

**Figure 2:**
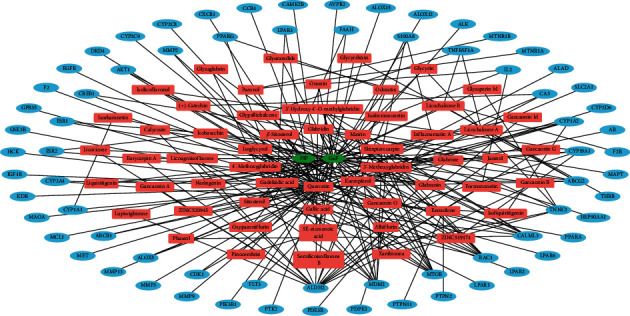
The herbal medicine-active chemical compound-target network of Jakyak-Gamcho decoction. Green nodes, herbal medicines; red nodes, active chemical compounds; blue nodes, pain-related targets.

To perform network pharmacology-based analysis of the pharmacological features of JGd, we constructed an herbal medicine-active chemical compound-target (H-C-T) network composed of 129 nodes (two herbal medicines, 57 active chemical compounds, and 70 pain-associated targets) and 217 links (Figure 2 and Supplementary Table S3) using comprehensive information regarding the herbal drug. We found that quercetin (number of targets = 36) and kaempferol (number of targets = 11) have relatively many targets (Figure 2 and Supplementary Table S3), implying that they might be important active compounds for the therapeutic activity of JGd. In addition, 27 human genes/proteins were found to be targeted by two or more active chemical compounds of JGd (Figure 2), suggesting a polypharmacological mechanism.

**Figure 3:**
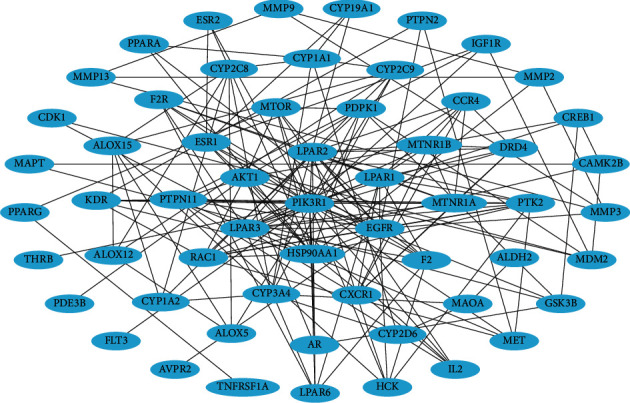
The protein-protein interaction network for the pain-related targets of Jakyak-Gamcho decoction. Blue nodes, pain-related targets.

To investigate the biological interaction relationship between the JGd targets, we generated a PPI network (58 nodes and 174 links) comprising the targets (Figure 3). Next, we searched for hubs, specific nodes with a high degree in the network that are shown to have crucial biological functions and promising therapeutic potential [129, 130]. In the analysis, hubs were determined as nodes for which the degree was greater than or equal to twice the average node degree of the network [131, 132]. The results showed that PIK3R1 (degree = 25), HSP90AA1 (degree = 15), EGFR (degree = 14), AKT1 (degree = 13), LPAR1 (degree = 13), LPAR2 (degree = 13), and LPAR3 (degree = 13) were hubs (Figure 3), implying that they might be the key targets responsible for the analgesic activity of JGd. These hubs were shown to be involved in the regulation of pain-related processes and could function as potent targets to induce analgesic effects. The *PIK3R1* gene was suggested to have the potential to function as a pain-related regulator according to the genetic interaction analysis [133], and its expression level might be associated with osteoarthritis pathogenesis [134]. Upregulation of the *HSP90AA1* gene was observed in patients with fibromyalgia [135–137], and pharmacological inhibition of heat shock protein 90 (HSP90; encoded by *HSP90AA1*) was shown to alleviate monoarthritis-induced pain [138]. The activation of epidermal growth factor receptor (EGFR; encoded by *EGFR*) and AKT (encoded by *AKT1*) is associated with the development and enhancement of diverse types of pain, and their therapeutic modulation might be associated with analgesic properties [139–156]. Lysophosphatidic acid receptor 1 (encoded by *LPAR1*) activity is involved in pain behavior arising from bone cancer, inflammation, diabetes, and neuropathy, and its pharmacological or genetic ablation might reduce the pain response [157–165]. Lysophosphatidic acid receptor 3 (encoded by *LPAR3*) plays crucial roles in the development and maintenance of neuropathic pain, and its blockade exerts analgesic effects [163, 166, 167].

We further assessed the CIs of the active chemical compounds of JGd to assess their pharmacological contribution to the analgesic effect of the herbal drug as described earlier [81, 168]. As a result, quercetin was shown to have the highest CI (91.83%) (Supplementary Figure S1), which suggests that this chemical compound might be the primary contributor to the analgesic activity of JGd.

Together, these data indicate the system-level pharmacological properties of the analgesic activity of JGd.

### 3.4. Functional Enrichment Investigation of Jakyak-Gamcho Decoction Networks

To investigate the molecular mechanisms underlying the analgesic effect of JGd, we carried out GO enrichment analysis of the targets. As a result, the JGd targets were enriched in GO terms involved in the modulation of a variety of biological activities, such as calcium- and cytokine-mediated signalings, calcium ion concentration and homeostasis, cellular behaviors of muscle and neuronal cells, inflammatory response, and response to chemical, cytokine, drug, and oxidative stress (Supplementary Figure S2), which are in accordance with the previously reported molecular mechanisms of the herbal drug [40, 41, 44, 46, 49, 55, 58–60, 169–172]. In addition, GeneMANIA analysis indicated that the JGd targets might functionally interact via diverse mechanisms (Supplementary Figure S3), implying the similarity in their pharmacological roles.

**Figure 4:**
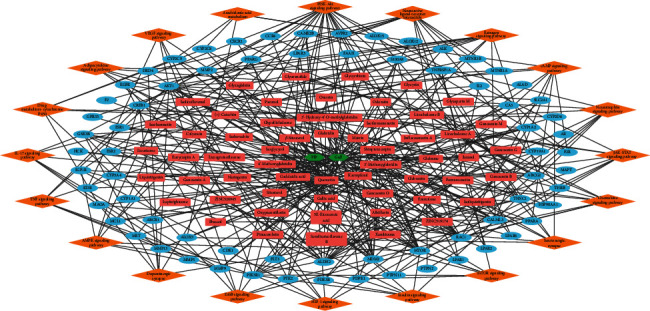
The herbal medicine-active chemical compound-target-pathway network of Jakyak-Gamcho decoction. Green nodes, herbal medicines; red nodes, active chemical compounds; blue nodes, pain-related targets; orange nodes, signaling pathways.

Because various signaling pathways were reported to be associated with the initiation, transmission, perception, and maintenance of pain [12, 14, 20, 144, 155, 173–186], we carried out pathway enrichment analysis. We found that the JGd targets were enriched in the following signalings: “PI3K-Akt signaling pathway,” “Neuroactive ligand-receptor interaction,” “Estrogen signaling pathway,” “cAMP signaling pathway,” “Chemokine signaling pathway,” “JAK-STAT signaling pathway,” “Neurotrophin signaling pathway,” “AMPK signaling pathway,” “Dopaminergic synapse,” “ErbB signaling pathway,” “HIF-1 signaling pathway,” “Insulin signaling pathway,” “mTOR signaling pathway,” “Serotonergic synapse,” “Adipocytokine signaling pathway,” “Drug metabolism - cytochrome P450,” “IL-17 signaling pathway,” “TNF signaling pathway,” “Arachidonic acid metabolism,” and “VEGF signaling pathway” (Figure 4 and Supplementary Figure S2). These signalings are well-known pain-regulating pathways and function as therapeutic targets of analgesic and pain-relieving drugs. The activities of adenosine monophosphate-activated kinase (AMPK), ErbB, mammalian target of rapamycin (mTOR), phosphoinositide 3-kinase (PI3K)-Akt, tumor necrosis factor (TNF), or vascular endothelial growth factor (VEGF) signaling pathways are involved with the development and maintenance processes of various types of pathological pain, and their functional modulation might relieve neuropathic, nociceptive, and bone cancer pain [144, 149, 150, 155, 156, 187–226]. Furthermore, the activity of PI3K-Akt and the adipocytokine pathway further correlates with the severity of neuropathic and inflammatory pain, and their targeting agents exert analgesic effects [227–229]. The estrogen pathway serves as a modulator of the processing and sensitivity of visceral and mechanical pain responses [230–235]. Previous studies have shown the involvement of cyclic adenosine monophosphate (cAMP), chemokine, Janus kinase- (JAK-) signal transducer and activator of transcription (STAT), neurotrophin, and hypoxia-inducible factor (HIF) pathways in the initiation and persistence of inflammatory, cancer, and neuropathic pain, as well as their role as pharmacological mediators of analgesic approaches [141, 224, 236–265]. The impaired regulation of insulin signaling might promote the development of and pain sensation with diabetic neuropathy, which can be alleviated by its functional restoration [266–269]. The interleukin- (IL-) 17 pathway plays a crucial role in cellular mechanisms of pain pathogenesis and maintenance in various diseases including multiple sclerosis, prostatitis, intervertebral disk degeneration, femoral head osteonecrosis, and neuropathy; its inhibition might block the generation and persistence of pain [270–282]. Arachidonic acid metabolism is associated with the generation and secretion of diverse biomolecular substances responsible for the induction of inflammation and pain, and it is mainly involved in the mechanisms of action of NSAIDs [283–288]. Moreover, the serotonergic and dopaminergic synapse pathways are key neurotransmitters responsible for modulating the intensity and duration of pain, and their therapeutic interventions have been shown to attenuate pain behaviors [289–292].

Collectively, these results demonstrate the molecular- and pathway-level mechanisms underlying the analgesic activity of JGd.

### 3.5. Molecular Docking Evaluation

**Figure 5:**
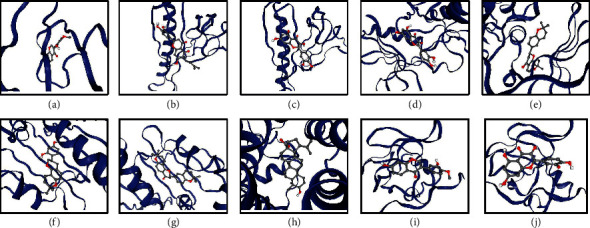
Molecular docking analysis of interactions between the active chemical compounds of Jakyak-Gamcho decoction and hub targets. (a) Calycosin-AKT1 (score = −7.5). (b) Gancaonin O-AKT1 (score = −5.7). (c) Quercetin-AKT1 (score = −6.4). (d) Quercetin-EGFR (score = −8.0). (e) Xambioona-EGFR (score = −10.5). (f) Glabridin-HSP90AA1 (score = −8.3). (g) Xambioona-HSP90AA1 (score = −9.1). (h) Mairin-LPAR1 (score = −8.4). (i) Calycosin-PIK3R1 (score = −7.9). (j) Quercetin-PIK3R1 (score = −6.3).

To investigate the binding potential of the chemical compounds of JGd components for the targets, we evaluated their molecular docking activity. As a result, 95.09% of the binding interactions between the active chemical components of JGd and the hub targets was found to have docking scores equal to or lower than −5.0 (Figure 5 and Supplementary Table S4), indicating their therapeutic binding potential. Of note, the protein structures for LPAR2 and LPAR3 were unavailable in the RCSB Protein Databank [110]; therefore, they were excluded from the analysis.

## 4. Discussion

Herbal medicines are increasingly being acknowledged as effective analgesic and pain-relieving agents owing to their promising therapeutic activity with fewer side effects [29–36]. JGd is a well-known herbal drug that alleviates pain induced by multiple diseases such as peripheral neuropathy, myalgia, arthralgia, and diabetes [30, 37–40, 44, 46–48], and it is one of the most frequently prescribed oral analgesics in East Asia [56]. Previous studies have attempted network pharmacology analyses to investigate the mechanisms underlying JGd for the treatment of osteoarthritis and Parkinson's disease [293, 294]; however, its network-perspective analgesic properties have not been fully elucidated. Therefore, this network pharmacology study attempted to investigate system-level mechanisms that underlie the analgesic activity of JGd. The ADME evaluation and network pharmacology investigation identified 57 active chemical compounds in JGd and their 70 pain-associated human molecular targets. Further enrichment analysis indicated that JGd targets were enriched with GO terms related to the modulation of biological activities, involving calcium- and cytokine-mediated signalings, calcium ion concentration and homeostasis, cellular behaviors of muscle and neuronal cells, inflammatory response, and response to chemical, cytokine, drug, and oxidative stress, consistent with the previously reported molecular mechanisms of the herbal drug [40, 41, 44, 46, 49, 55, 58–60, 169–172]. We further showed that JGd might target various pain signalings to exert its analgesic and pain-relieving effects, which involve the PI3K-Akt, estrogen, ErbB, neurotrophin, neuroactive ligand-receptor interaction, HIF-1, serotonergic synapse, JAK-STAT, and cAMP pathways.

The analgesic activity of the chemical components of JGd has been previously reported. (+)-Catechin and pinocembrin produce analgesic, antineuropathy, and antinociceptive effects [295–297]. Albiflorin might play a pharmacological role as an analgesic, antineuropathy, and antinociceptive compound that can reduce pain intensity via the functional modulation of calcium channels, mitogen-activated protein kinase (MAPK) pathways, and various cytokines and chemokines [127, 298]. Moreover, formononetin, glabridin, glycyrrhizin, and paeonol exhibit anti-inflammatory, antinociceptive, and analgesic activities by inhibiting the generation of inflammatory cytokines and signaling molecules, thereby attenuating the pain responses [117, 120, 299–302]. Gallic acid could also have potential anti-inflammatory, antioxidant, and neuroprotective effects that could improve neuropathic pain, neuronal damage, and injury [119, 303, 304]. Glycyrrhizin and naringenin reduce inflammatory and neuropathic pain-like behaviors by modulating the secretion of inflammation-associated cytokines and mediators, as well as the activities of cyclic guanosine monophosphate (cGMP) and nuclear factor kappa-light-chain-enhancer of activated B cells (NF-*κ*B) signalings [120, 301, 305–314]. Isoliquiritigenin was reported to possess analgesic, antispasmodic, and relaxant properties [315, 316]. Isorhamnetin ameliorates the pain intensity of diabetic neuropathy via its neuroprotective, antioxidative, and anti-inflammatory effects [317]. Kaempferol shows anti-inflammatory, antioxidant, and analgesic effects, which relieve the pain symptoms of gastritis, pancreatitis, and diabetic neuropathy [318–320]. In addition, liquiritigenin might suppress neuropathic pain by improving thermal, cold, and mechanical hyperalgesia [116]. Mairin (betulinic acid) has been shown to exert anti-inflammatory, antinociceptive, antipyretic, and analgesic effects, thereby alleviating visceral pain and chemotherapy-, infection-, and diabetes-associated neuropathies [321–326]. Quercetin reduces pain arising from inflammation, cancer, chronic prostatitis/chronic pelvic pain syndrome, arthritis, and muscle injury by inhibiting the induction of oxidative stress and activating inflammatory and adrenergic pathways, neurotransmitters, and cytokines [327–334]. In addition, quercetin further modulates the activity of a variety of pathways, including Toll-like receptor, mTOR, protein kinase C*ε* (PKC*ε*)-TRPV1, p70 ribosomal S6 kinase (p70S6K), and P2X_4_ receptor signalings, as well as oxidative stress- and inflammation-associated mediators to exert its analgesic effects against diverse types of neuropathic pain [214, 335–347]. *β*-Sitosterol shows analgesic, antinociceptive, and anti-inflammatory activities [348–353]. These studies regarding the chemical components of JGd provide the pharmacological basis for the analgesic activities of this herbal drug.

Based on the network pharmacological analyses, the following studies would contribute to the improvement of herbal drug therapies: (i) an assessment of the therapeutic efficacy of JGd analgesic activity in specific diseases that are associated with distinct types of pain, such as cancer, osteoarthritis, myalgia, arthralgia, and diabetes; (ii) a comprehensive exploration of the system-level mechanisms of analgesic properties of the herbal drug from diverse pharmacological perspectives, involving antinociceptive, anti-inflammatory, muscle relaxant, and antipyretic effects; and (iii) an investigation of the safety and effectiveness of combined treatment with JGd and widely used analgesic agents, including celecoxib, tramadol, and acetaminophen [24, 56, 354].

To conclude, we investigated the systems' perspective pharmacological properties of JGd, a widely prescribed analgesic herbal drug [56]. Based on the network pharmacological approach, we investigated 57 active chemical compounds and their 70 pain-related targets responsible for the analgesic activity of JGd. The targets of JGd were associated with the modulation of biological functions such as calcium- and cytokine-mediated signalings, calcium ion concentration and homeostasis, cellular behaviors of muscle and neuronal cells, inflammatory response, and response to chemical, cytokine, drug, and oxidative stress, which suggests the molecular mechanisms of JGd treatment. In addition, the enrichment analysis indicated that the targets are involved in various pathways that are associated with the pathophysiology of pain, including the PI3K-Akt, estrogen, ErbB, neurotrophin, neuroactive ligand-receptor interaction, HIF-1, serotonergic synapse, JAK-STAT, and cAMP pathways. The overall data offer a novel systematic view of the polypharmacological characteristics of herbal drugs and a mechanistic basis for their clinical implications for pain treatment.

## Data Availability

The data used to support the findings of this study are included within the article and supplementary materials file.

## Supplementary Materials

Supplementary Materials Supplementary Figure S1: contribution index analysis for the analgesic activity of active chemical compounds of Jakyak-Gamcho decoction. Supplementary Figure S2: functional enrichment analyses for the pain-associated targets of Jakyak-Gamcho decoction. Supplementary Figure S3: functional interaction analysis of the pain-associated targets of Jakyak-Gamcho decoction. Supplementary Table S1: list of the chemical compounds contained in Jakyak-Gamcho decoction. Supplementary Table S2: list of the active chemical compounds contained in Jakyak-Gamcho decoction. Supplementary Table S3: list of the targets of active chemical compounds of Jakyak-Gamcho decoction. Supplementary Table S4: docking scores of the active chemical compounds of Jakyak-Gamcho decoction with the hub targets.

## References

[1] Goudas L. C., Bloch R., Gialeli-Goudas M., Lau J., Carr D. B. (2005). The epidemiology of cancer pain. *Cancer Investigation*.

[2] Macfarlane G. J. (2016). The epidemiology of chronic pain. *Pain*.

[3] Russo M. M., Sundaramurthi T. (2019). An overview of cancer pain: epidemiology and pathophysiology. *Seminars in Oncology Nursing*.

[4] Smith B. H., Torrance N. (2012). Epidemiology of neuropathic pain and its impact on quality of life. *Current Pain and Headache Reports*.

[5] Stucky C. L., Gold M. S., Zhang X. (2001). Mechanisms of pain. *Proceedings of the National Academy of Sciences*.

[6] Wolkerstorfer A., Handler N., Buschmann H. (2016). New approaches to treating pain. *Bioorganic & Medicinal Chemistry Letters*.

[7] Butera J. A. (2007). Current and emerging targets to treat neuropathic pain. *Journal of Medicinal Chemistry*.

[8] Chakravarty A., Sen A. (2010). Migraine, neuropathic pain and nociceptive pain: towards a unifying concept. *Medical Hypotheses*.

[9] Colloca L., Ludman T., Bouhassira D. (2017). Neuropathic pain. *Nature Reviews Disease Primers*.

[10] Epstein J. B., Wilkie D. J., Fischer D. J., Kim Y.-O., Villines D. (2009). Neuropathic and nociceptive pain in head and neck cancer patients receiving radiation therapy. *Head & Neck Oncology*.

[11] Freynhagen R., Parada H. A., Calderon-Ospina C. A. (2019). Current understanding of the mixed pain concept: a brief narrative review. *Current Medical Research and Opinion*.

[12] Hunt S. P., Mantyh P. W. (2001). The molecular dynamics of pain control. *Nature Reviews Neuroscience*.

[13] Jehangir A., Abdallah R. T., Parkman H. P. (2019). Characterizing abdominal pain in patients with gastroparesis into neuropathic and nociceptive components. *Journal of Clinical Gastroenterology*.

[14] Jensen M. P., Day M. A., Miró J. (2014). Neuromodulatory treatments for chronic pain: efficacy and mechanisms. *Nature Reviews Neurology*.

[15] Mantyh P. W. (2006). Cancer pain and its impact on diagnosis, survival and quality of life. *Nature Reviews Neuroscience*.

[16] Mantyh P. W., Clohisy D. R., Koltzenburg M., Hunt S. P. (2002). Molecular mechanisms of cancer pain. *Nature Reviews Cancer*.

[17] Morlion B. (2011). Pharmacotherapy of low back pain: targeting nociceptive and neuropathic pain components. *Current Medical Research and Opinion*.

[18] Nijs J., Leysen L., Adriaenssens N. (2016). Pain following cancer treatment: guidelines for the clinical classification of predominant neuropathic, nociceptive and central sensitization pain. *Acta Oncologica*.

[19] Omoigui S. (2007). The biochemical origin of pain—proposing a new law of pain: the origin of all pain is inflammation and the inflammatory response. Part 1 of 3—a unifying law of pain. *Medical Hypotheses*.

[20] Thakur M., Dickenson A. H., Baron R. (2014). Osteoarthritis pain: nociceptive or neuropathic?. *Nature Reviews Rheumatology*.

[21] Yoon S. Y., Oh J. (2018). Neuropathic cancer pain: prevalence, pathophysiology, and management. *The Korean Journal of Internal Medicine*.

[22] Crofford L. J. (2010). Adverse effects of chronic opioid therapy for chronic musculoskeletal pain. *Nature Reviews Rheumatology*.

[23] Fine P. G. (2002). Analgesia issues in palliative care: bone pain, controlled release opioids, managing opioid-induced constipation and nifedipine as an analgesic. *Journal of Pain & Palliative Care Pharmacotherapy*.

[24] Kim S.-J., Seo J. T. (2020). Selection of analgesics for the management of acute and postoperative dental pain: a mini-review. *Journal of Periodontal & Implant Science*.

[25] Malfait A.-M., Schnitzer T. J. (2013). Towards a mechanism-based approach to pain management in osteoarthritis. *Nature Reviews Rheumatology*.

[26] Nelson A. D., Camilleri M. (2016). Opioid-induced constipation: advances and clinical guidance. *Therapeutic Advances in Chronic Disease*.

[27] Tolman K. G. (1998). Hepatotoxicity of non-narcotic analgesics. *The American Journal of Medicine*.

[28] Yekkirala A. S., Roberson D. P., Bean B. P., Woolf C. J. (2017). Breaking barriers to novel analgesic drug development. *Nature Reviews Drug Discovery*.

[29] Chen L., Michalsen A. (2017). Management of chronic pain using complementary and integrative medicine. *BMJ*.

[30] Hyodo T., Taira T., Takemura T. (2006). Immediate effect of shakuyaku-kanzo-to on muscle cramp in hemodialysis patients. *Nephron Clinical Practice*.

[31] Kono T., Mamiya N., Chisato N. (2011). Efficacy of goshajinkigan for peripheral neurotoxicity of oxaliplatin in patients with advanced or recurrent colorectal cancer. *Evid Based Complement Alternat Med*.

[32] Luo Y., Wang C.-Z., Sawadogo R., Tan T., Yuan C.-S. (2020). Effects of herbal medicines on pain management. *The American Journal of Chinese Medicine*.

[33] Nishioka M., Shimada M., Kurita N. (2011). The kampo medicine, goshajinkigan, prevents neuropathy in patients treated by FOLFOX regimen. *International Journal of Clinical Oncology*.

[34] Tawata M., Kurihara A., Nitta K., Iwase E., Gan N., Onaya T. (1994). The effects of goshajinkigan, a herbal medicine, on subjective symptoms and vibratory threshold in patients with diabetic neuropathy. *Diabetes Research and Clinical Practice*.

[35] Yanju B., Yang L., Hua B. (2014). A systematic review and meta-analysis on the use of traditional Chinese medicine compound kushen injection for bone cancer pain. *Supportive Care in Cancer*.

[36] Yuan C.-S., Mehendale S. R., Wang C.-Z. (2004). Effects of *Corydalis yanhusuo* and *Angelicae dahuricae* on cold pressor-induced pain in humans: a controlled trial. *The Journal of Clinical Pharmacology*.

[37] Fujii K., Okamoto S., Saitoh K. (2004). The efficacy of shakuyaku-kanzo-to for peripheral nerve dysfunction in paclitaxel combination chemotherapy for epithelial ovarian carcinoma. *Gan To Kagaku Ryoho*.

[38] Fujiwara H., Urabe T., Ueda K. (2000). Prevention of arthralgia and myalgia from paclitaxel and carboplatin combination chemotherapy with shakuyaku-kanzo-to. *Gan To Kagaku Ryoho*.

[39] Hidaka T., Shima T., Nagira K. (2009). Herbal medicine shakuyaku-kanzo-to reduces paclitaxel-induced painful peripheral neuropathy in mice. *European Journal of Pain*.

[40] Hinoshita F., Ogura Y., Suzuki Y. (2003). Effect of orally administered shao-yao-gan-cao-tang (shakuyaku-kanzo-to) on muscle cramps in maintenance hemodialysis patients: a preliminary study. *The American Journal of Chinese Medicine*.

[41] Lee K. K., Omiya Y., Yuzurihara M., Kase Y., Kobayashi H. (2013). Antispasmodic effect of shakuyakukanzoto extract on experimental muscle cramps in vivo: role of the active constituents of Glycyrrhizae radix. *Journal of Ethnopharmacology*.

[42] Liu J. j., Cheng Y., Shao Y. y. (2019). Comparative pharmacokinetics and metabolites study of seven major bioactive components of shaoyao-gancao decoction in normal and polycystic ovary syndrome rats by ultra high pressure liquid chromatography with tandem mass spectrometry. *Journal of Separation Science*.

[43] Shao Y. Y., Chang Z. P., Cheng Y. (2019). Shaoyao-Gancao decoction alleviated hyperandrogenism in a letrozole-induced rat model of polycystic ovary syndrome by inhibition of NF-*κ*B activation. *Bioscience Reports*.

[44] Sui F., Zhou H.-Y., Meng J. (2016). A Chinese herbal decoction, shaoyao-gancao tang, exerts analgesic effect by down-regulating the TRPV1 channel in a rat model of arthritic pain. *The American Journal of Chinese Medicine*.

[45] Takahashi K., Kitao M. (1994). Effect of TJ-68 (shakuyaku-kanzo-to) on polycystic ovarian disease. *International Journal of Fertility and Menopausal Studies*.

[46] Tsuji S., Yasuda K., Sumi G. (2012). Shakuyaku-kanzo-to inhibits smooth muscle contractions of human pregnant uterine tissue in vitro. *Journal of Obstetrics and Gynaecology Research*.

[47] Yamamoto K., Hoshiai H., Noda K. (2001). Effects of shakuyaku-kanzo-to on muscle pain from combination chemotherapy with paclitaxel and carboplatin. *Gynecologic Oncology*.

[48] Yoshida T., Sawa T., Ishiguro T., Horiba A., Minatoguchi S., Fujiwara H. (2009). The efficacy of prophylactic shakuyaku-kanzo-to for myalgia and arthralgia following carboplatin and paclitaxel combination chemotherapy for non-small cell lung cancer. *Supportive Care in Cancer*.

[49] Chen I. C., Lin T. H., Hsieh Y. H. (2018). Formulated Chinese medicine shaoyao gancao tang reduces tau aggregation and exerts neuroprotection through anti-oxidation and anti-inflammation. *Oxid Med Cell Longev*.

[50] Fujinami H., Kajiura S., Ando T., Mihara H., Hosokawa A., Sugiyama T. (2015). Direct spraying of shakuyakukanzoto onto the duodenal papilla: a novel method for preventing pancreatitis following endoscopic retrograde cholangiopancreatography. *Digestion*.

[51] Kaifuchi N., Omiya Y., Kushida H., Fukutake M., Nishimura H., Kase Y. (2015). Effects of shakuyakukanzoto and its absorbed components on twitch contractions induced by physiological Ca^2+^ release in rat skeletal muscle. *Journal of Natural Medicines*.

[52] Sakai Y., Tsuyuguchi T., Ishihara T. (2009). Confirmation of the antispasmodic effect of shakuyaku-kanzo-to (TJ-68), a Chinese herbal medicine, on the duodenal wall by direct spraying during endoscopic retrograde cholangiopancreatography. *Journal of Natural Medicines*.

[53] Shen L., Cong W.-J., Lin X. (2012). Characterization using LC/MS of the absorption compounds and metabolites in rat plasma after oral administration of a single or mixed decoction of shaoyao and gancao. *Chemical and Pharmaceutical Bulletin*.

[54] Shen L., Hu R.-w., Lin X. (2012). Pharmacokinetics of characteristic effective ingredients from individual and combination shaoyao and gancao treatement in rats using HPLC fingerprinting. *European Journal of Drug Metabolism and Pharmacokinetics*.

[55] Zhang Y., Jia X., Yang J. (2016). Effects of shaoyao-gancao decoction on infarcted cerebral cortical neurons: suppression of the inflammatory response following cerebral ischemia-reperfusion in a rat model. *BioMed Research International*.

[56] Ushida T., Matsui D., Inoue T. (2019). Recent prescription status of oral analgesics in Japan in real-world clinical settings: retrospective study using a large-scale prescription database. *Expert Opinion on Pharmacotherapy*.

[57] Feng L.-M., Chen Y.-Y., Xu D.-Q. (2020). An integrated strategy for discovering effective components of shaoyao gancao decoction for treating neuropathic pain by the combination of partial least-squares regression and multi-index comprehensive method. *Journal of Ethnopharmacology*.

[58] Omiya Y., Suzuki Y., Yuzurihara M. (2005). Antinociceptive effect of shakuyakukanzoto, a kampo medicine, in diabetic mice. *Journal of Pharmacological Sciences*.

[59] Zhang J., Lv C., Wang H.-n., Cao Y. (2013). Synergistic interaction between total glucosides and total flavonoids on chronic constriction injury induced neuropathic pain in rats. *Pharmaceutical Biology*.

[60] Zheng D., Zhang J., Wang R., Lu C., Guo X., Wang H. J. (2013). Administration of the influence of shaoyao gancao decoction extracts on IL-6 IL-1*β* and TNF-*α* in the chronic constriction injury rat model of neuropathic pain. *Chinese Archives of Traditional Chinese Medicine*.

[61] Poornima P., Kumar J. D., Zhao Q., Blunder M., Efferth T. (2016). Network pharmacology of cancer: from understanding of complex interactomes to the design of multi-target specific therapeutics from nature. *Pharmacological Research*.

[62] Lee W. Y., Lee C. Y., Kim Y. S., Kim C. E. (2019). The methodological trends of traditional herbal medicine employing network pharmacology. *Biomolecules*.

[63] Lee H. S., Lee I. H., Park S. I., Lee D. Y. (2020). Network pharmacology-based investigation of the system-level molecular mechanisms of the hematopoietic activity of Samul-Tang, a traditional Korean herbal formula. *Evidence-Based Complementary and Alternative Medicine*.

[64] He R., Ou S., Chen S., Ding S. (2020). Network pharmacology-based study on the molecular biological mechanism of action for compound kushen injection in anti-cancer effect. *Medical Science Monitor*.

[65] Mi J. L., Liu C., Xu M., Wang R. S. (2020). Network pharmacology to uncover the molecular mechanisms of action of LeiGongTeng for the treatment of nasopharyngeal carcinoma. *Medical Science Monitor Basic Research*.

[66] Wang Y., Dong B., Xue W. (2020). Anticancer effect of radix astragali on cholangiocarcinoma in vitro and its mechanism via network pharmacology. *Medical Science Monitor*.

[67] Xu T., Wang Q., Liu M. (2020). A network pharmacology approach to explore the potential mechanisms of huangqin-baishao herb pair in treatment of cancer. *Medical Science Monitor*.

[68] Zhang S. Q., Xu H. B., Zhang S. J., Li X. Y. (2020). Identification of the active compounds and significant pathways of *Artemisia annua* in the treatment of non-small cell lung carcinoma based on network pharmacology. *Medical Science Monitor*.

[69] Hu Z., Yang M., Yang L. (2020). Network pharmacology-based identification of the mechanisms of Shen-Qi compound formula in treating diabetes mellitus. *Evidence-Based Complementary and Alternative Medicine*.

[70] Jiang Y., Zhong M., Long F., Yang R. (2020). Deciphering the active ingredients and molecular mechanisms of *Tripterygium hypoglaucum* (Levl.) hutch against rheumatoid arthritis based on network pharmacology. *Evidence-Based Complementary and Alternative Medicine*.

[71] Li D. H., Su Y. F., Sun C. X., Fan H. F., Gao W. J. (2020). A network pharmacology-based identification study on the mechanism of Xiao-Xu-Ming decoction for cerebral ischemic stroke. *Evidence-Based Complementary and Alternative Medicine*.

[72] Liu W., Fan Y., Tian C. (2020). Deciphering the molecular targets and mechanisms of HGWD in the treatment of rheumatoid arthritis via network pharmacology and molecular docking. *Evidence-Based Complementary and Alternative Medicine*.

[73] Qian H., Jin Q., Liu Y. (2020). Study on the multitarget mechanism of sanmiao pill on gouty arthritis based on network pharmacology. *Evidence-Based Complementary and Alternative Medicine*.

[74] Ren B., Tan L., Xiong Y. (2020). Integrated analysis of the mechanisms of Da-Chai-Hu decoction in type 2 diabetes mellitus by a network pharmacology approach. *Evidence-Based Complementary and Alternative Medicine*.

[75] Wang W., Zhang Y., Luo J., Wang R., Tang C., Zhang Y. (2020). Virtual screening technique used to estimate the mechanism of *Adhatoda vasica* nees for the treatment of rheumatoid arthritis based on network pharmacology and molecular docking. *Evidence-Based Complementary and Alternative Medicine*.

[76] Xiao K., Li K., Long S., Kong C., Zhu S. (2020). Potential molecular mechanisms of Chaihu-Shugan-San in treatment of breast cancer based on network pharmacology. *Evidence-Based Complementary and Alternative Medicine*.

[77] Yang K., Zeng L., Ge J. (2018). Exploring the pharmacological mechanism of Danzhi Xiaoyao powder on ER-positive breast cancer by a network pharmacology approach. *Evidence-Based Complementary and Alternative Medicine*.

[78] Zhang C., Liao Y., Liu L. (2020). A network pharmacology approach to investigate the active compounds and mechanisms of musk for ischemic stroke. *Evidence-Based Complementary and Alternative Medicine*.

[79] Zhou J., Wang Q., Xiang Z. (2019). Network pharmacology analysis of traditional Chinese medicine formula *Xiao Ke Yin Shui* treating type 2 diabetes mellitus. *Evidence-Based Complementary and Alternative Medicine*.

[80] Ru J., Li P., Wang J. (2014). TCMSP: a database of systems pharmacology for drug discovery from herbal medicines. *Journal of Cheminformatics*.

[81] Yue S. J., Xin L. T., Fan Y. C. (2017). Herb pair Danggui-Honghua: mechanisms underlying blood stasis syndrome by system pharmacology approach. *Scientific Reports*.

[82] Wang C. K., Craik D. J. (2016). Cyclic peptide oral bioavailability: lessons from the past. *Biopolymers*.

[83] Kono Y., Iwasaki A., Matsuoka K., Fujita T. (2016). Effect of mechanical agitation on cationic liposome transport across an unstirred water layer in caco-2 cells. *Biological & Pharmaceutical Bulletin*.

[84] Volpe D. A. (2008). Variability in caco-2 and MDCK cell-based intestinal permeability assays. *Journal of Pharmaceutical Sciences*.

[85] Garcia M. N., Flowers C., Cook J. D. (1996). The caco-2 cell culture system can be used as a model to study food iron availability. *The Journal of Nutrition*.

[86] Li Y., Zhang J., Zhang L. (2015). Systems pharmacology to decipher the combinational anti-migraine effects of Tianshu formula. *Journal of Ethnopharmacology*.

[87] Zhang J., Li Y., Chen X., Pan Y., Zhang S., Wang Y. (2014). Systems pharmacology dissection of multi-scale mechanisms of action for herbal medicines in stroke treatment and prevention. *PLoS One*.

[88] Lee A. Y., Park W., Kang T.-W., Cha M. H., Chun J. M. (2018). Network pharmacology-based prediction of active compounds and molecular targets in Yijin-Tang acting on hyperlipidaemia and atherosclerosis. *Journal of Ethnopharmacology*.

[89] Wang X., Shen Y., Wang S. (2017). PharmMapper 2017 update: a web server for potential drug target identification with a comprehensive target pharmacophore database. *Nucleic Acids Research*.

[90] Szklarczyk D., Santos A., von Mering C., Jensen L. J., Bork P., Kuhn M. (2016). Stitch 5: augmenting protein-chemical interaction networks with tissue and affinity data. *Nucleic Acids Research*.

[91] Daina A., Michielin O., Zoete V. (2019). SwissTargetPrediction: updated data and new features for efficient prediction of protein targets of small molecules. *Nucleic Acids Research*.

[92] Keiser M. J., Roth B. L., Armbruster B. N., Ernsberger P., Irwin J. J., Shoichet B. K. (2007). Relating protein pharmacology by ligand chemistry. *Nature Biotechnology*.

[93] Yu H., Chen J., Xu X. (2012). A systematic prediction of multiple drug-target interactions from chemical, genomic, and pharmacological data. *PLoS One*.

[94] Zheng C., Guo Z., Huang C. (2015). Large-scale direct targeting for drug repositioning and discovery. *Scientific Reports*.

[95] Piñero J., Bravo À., Queralt-Rosinach N. (2017). DisGeNET: a comprehensive platform integrating information on human disease-associated genes and variants. *Nucleic Acids Research*.

[96] Zhu F., Han B., Kumar P. (2010). Update of TTD: therapeutic target database. *Nucleic Acids Research*.

[97] Safran M., Dalah I., Alexander J. (2010). GeneCards version 3: the human gene integrator. *Database*.

[98] Davis A. P., Grondin C. J., Johnson R. J. (2019). The comparative toxicogenomics database: update 2019. *Nucleic Acids Research*.

[99] Yu W., Gwinn M., Clyne M., Yesupriya A., Khoury M. J. (2008). A navigator for human genome epidemiology. *Nature Genetics*.

[100] Amberger J. S., Bocchini C. A., Schiettecatte F., Scott A. F., Hamosh A. (2015). OMIM.org: online Mendelian inheritance in man (OMIM®), an online catalog of human genes and genetic disorders. *Nucleic Acids Research*.

[101] Whirl-Carrillo M., McDonagh E. M., Hebert J. M. (2012). Pharmacogenomics knowledge for personalized medicine. *Clinical Pharmacology & Therapeutics*.

[102] Wishart D. S., Feunang Y. D., Guo A. C. (2018). DrugBank 5.0: a major update to the DrugBank database for 2018. *Nucleic Acids Research*.

[103] Szklarczyk D., Gable A. L., Lyon D. (2019). STRING v11: protein-protein association networks with increased coverage, supporting functional discovery in genome-wide experimental datasets. *Nucleic Acids Research*.

[104] Shannon P., Markiel A., Ozier O. (2003). Cytoscape: a software environment for integrated models of biomolecular interaction networks. *Genome Research*.

[105] Barabási A.-L., Oltvai Z. N. (2004). Network biology: understanding the cell’s functional organization. *Nature Reviews Genetics*.

[106] Raudvere U., Kolberg L., Kuzmin I. (2019). g:Profiler: a web server for functional enrichment analysis and conversions of gene lists (2019 update). *Nucleic Acids Research*.

[107] Kanehisa M., Goto S. (2000). KEGG: kyoto encyclopedia of genes and genomes. *Nucleic Acids Research*.

[108] Montojo J., Zuberi K., Rodriguez H., Bader G. D., Morris Q. (2014). GeneMANIA: fast gene network construction and function prediction for Cytoscape. *F1000 Research*.

[109] Kim S., Chen J., Cheng T. (2019). PubChem 2019 update: improved access to chemical data. *Nucleic Acids Research*.

[110] Burley S. K., Berman H. M., Bhikadiya C. (2019). RCSB protein data bank: biological macromolecular structures enabling research and education in fundamental biology, biomedicine, biotechnology and energy. *Nucleic Acids Research*.

[111] Trott O., Olson A. J. (2010). AutoDock vina: improving the speed and accuracy of docking with a new scoring function, efficient optimization, and multithreading. *Journal of Computational Chemistry*.

[112] Zhuang Z., Wen J., Zhang L. (2020). Can network pharmacology identify the anti-virus and anti- inflammatory activities of shuanghuanglian oral liquid used in Chinese medicine for respiratory tract infection?. *European Journal of Integrative Medicine*.

[113] Zhang M., Yuan Y., Zhou W. (2020). Network pharmacology analysis of Chaihu Lizhong Tang treating non-alcoholic fatty liver disease. *Computational Biology and Chemistry*.

[114] Boisnic S., Ben Slama L., Branchet-Gumila M. C., Watts M., d’Arros G. (2010). Effet anti-inflammatoire de l’enoxolone dans un modèle ex-vivo de muqueuse gingivale humaine. *Revue de Stomatologie et de Chirurgie Maxillo-Faciale*.

[115] Chae H.-S., Kang O.-H., Lee Y.-S. (2009). Inhibition of LPS-induced iNOS, COX-2 and inflammatory mediator expression by paeonol through the MAPKs inactivation in RAW 264.7 cells. *The American Journal of Chinese Medicine*.

[116] Chen L., Chen W., Qian X., Fang Y., Zhu N. (2014). Liquiritigenin alleviates mechanical and cold hyperalgesia in a rat neuropathic pain model. *Scientific Reports*.

[117] Chou T.-C. (2003). Anti-inflammatory and analgesic effects of paeonol in carrageenan-evoked thermal hyperalgesia. *British Journal of Pharmacology*.

[118] Dong L., Yin L., Zhang Y., Fu X., Lu J. (2017). Anti-inflammatory effects of ononin on lipopolysaccharide-stimulated RAW 264.7 cells. *Molecular Immunology*.

[119] Kaur S., Muthuraman A. (2019). Ameliorative effect of gallic acid in paclitaxel-induced neuropathic pain in mice. *Toxicology Reports*.

[120] Sun X., Zeng H., Wang Q. (2018). Glycyrrhizin ameliorates inflammatory pain by inhibiting microglial activation-mediated inflammatory response via blockage of the HMGB1-TLR4-NF-kB pathway. *Experimental Cell Research*.

[121] Wang Y., Xu C., Wang P. (2013). Pharmacokinetic comparisons of different combinations of shaoyao-gancao-decoction in rats: simultaneous determination of ten active constituents by HPLC-MS/MS. *Journal of Chromatography B*.

[122] Xu C.-H., Wang P., Wang Y. (2013). Pharmacokinetic comparisons of two different combinations of shaoyao-gancao decoction in rats: competing mechanisms between paeoniflorin and glycyrrhetinic acid. *Journal of Ethnopharmacology*.

[123] Yerra V. G., Kalvala A. K., Kumar A. (2017). Isoliquiritigenin reduces oxidative damage and alleviates mitochondrial impairment by SIRT1 activation in experimental diabetic neuropathy. *The Journal of Nutritional Biochemistry*.

[124] Yu C., Zhang Y., Gao K.-X. (2020). Serotonergically dependent antihyperalgesic and antiallodynic effects of isoliquiritin in a mouse model of neuropathic pain. *European Journal of Pharmacology*.

[125] Zhang L., Li D.-c., Liu L.-f. (2019). Paeonol: pharmacological effects and mechanisms of action. *International Immunopharmacology*.

[126] Zhang M.-T., Wang B., Jia Y.-N. (2017). Neuroprotective effect of liquiritin against neuropathic pain induced by chronic constriction injury of the sciatic nerve in mice. *Biomedicine & Pharmacotherapy*.

[127] Zhou J., Wang L., Wang J. (2016). Paeoniflorin and albiflorin attenuate neuropathic pain via MAPK pathway in chronic constriction injury rats. *Evidence-Based Complementary and Alternative Medicine*.

[128] Kim S.-A., Jang E.-S., Lee A., Lee S.-J., Kim J.-H. (2020). Anti-inflammatory and anti-oxidant effects of oxypaeoniflorin, paeoniflorin and *Paeonia lactiflora* cv. “red charm” flower petal extracts in macrophage cells. *Korean Journal of Plant Resources*.

[129] Cho D. Y., Kim Y. A., Przytycka T. M. (2012). Chapter 5: network biology approach to complex diseases. *PLoS Computational Biology*.

[130] Jeong H., Mason S. P., Barabási A.-L., Oltvai Z. N. (2001). Lethality and centrality in protein networks. *Nature*.

[131] Zhu J., Yi X., Zhang Y., Pan Z., Zhong L., Huang P. (2019). Systems pharmacology-based approach to comparatively study the independent and synergistic mechanisms of danhong injection and naoxintong capsule in ischemic stroke treatment. *Evidence-Based Complementary and Alternative Medicine*.

[132] Zhong J., Liu Z., Zhou X., Xu J. (2017). Synergic anti-pruritus mechanisms of action for the radix *Sophorae flavescentis* and fructus cnidii herbal pair. *Molecules*.

[133] Perkins J. R., Lees J., Antunes-Martins A. (2013). PainNetworks: a web-based resource for the visualisation of pain-related genes in the context of their network associations. *Pain*.

[134] Shi T., Shen X., Gao G. (2019). Gene expression profiles of peripheral blood monocytes in osteoarthritis and analysis of differentially expressed genes. *BioMed Research International*.

[135] Lukkahatai N., Majors B., Reddy S., Walitt B., Saligan L. N. (2013). Gene expression profiles of fatigued fibromyalgia patients with different categories of pain and catastrophizing: a preliminary report. *Nursing Outlook*.

[136] Lukkahatai N., Walitt B., Espina A., Wang D., Saligan L. N. (2015). Comparing genomic profiles of women with and without fibromyalgia. *Biological Research For Nursing*.

[137] Vincent A., Benzo R. P., Whipple M. O., McAllister S. J., Erwin P. J., Saligan L. N. (2013). Beyond pain in fibromyalgia: insights into the symptom of fatigue. *Arthritis Research & Therapy*.

[138] Nascimento D. S. M., Potes C. S., Soares M. L. (2018). Drug-induced HSP90 inhibition alleviates pain in monoarthritic rats and alters the expression of new putative pain players at the DRG. *Molecular Neurobiology*.

[139] Duan B., Liu D.-S., Huang Y. (2012). PI3-kinase/Akt pathway-regulated membrane insertion of acid-sensing ion channel 1a underlies BDNF-induced pain hypersensitivity. *Journal of Neuroscience*.

[140] Guan X., Fu Q., Xiong B. (2015). Activation of PI3K*γ*/Akt pathway mediates bone cancer pain in rats. *Journal of Neurochemistry*.

[141] Guan X.-H., Fu Q.-C., Shi D. (2015). Activation of spinal chemokine receptor CXCR3 mediates bone cancer pain through an Akt-ERK crosstalk pathway in rats. *Experimental Neurology*.

[142] Guedes R. P., Araújo A. S. R., Janner D., Belló-Klein A., Ribeiro M. F. M., Partata W. A. (2008). Increase in reactive oxygen species and activation of Akt signaling pathway in neuropathic pain. *Cellular and Molecular Neurobiology*.

[143] Hu B., Xu G., Zhang X. (2018). Paeoniflorin attenuates inflammatory pain by inhibiting microglial activation and Akt-NF-*κ*b signaling in the central nervous system. *Cellular Physiology and Biochemistry*.

[144] Jin D., Yang J.-p., Hu J.-h., Wang L.-n., Zuo J.-l. (2015). MCP-1 stimulates spinal microglia via PI3K/Akt pathway in bone cancer pain. *Brain Research*.

[145] Kersten C., Cameron M. G. (2012). Cetuximab alleviates neuropathic pain despite tumour progression. *BMJ Case Reports*.

[146] Kersten C., Cameron M. G., Laird B., Mjåland S. (2015). Epidermal growth factor receptor—inhibition (EGFR-I) in the treatment of neuropathic pain. *British Journal of Anaesthesia*.

[147] Li D., Chen H., Luo X.-H., Sun Y., Xia W., Xiong Y.-C. (2016). CX3CR1-mediated Akt1 activation contributes to the paclitaxel-induced painful peripheral neuropathy in rats. *Neurochemical Research*.

[148] Li S.-S., Zhang W.-S., Yang J.-L., Xiong Y.-C., Zhang Y.-Q., Xu H. (2013). Involvement of protein kinase B/Akt in analgesic effect of dexmedetomidine on neuropathic pain. *CNS Neuroscience & Therapeutics*.

[149] Li X., Yang S., Wang L. (2019). Resveratrol inhibits paclitaxel-induced neuropathic pain by the activation of PI3K/Akt and SIRT1/PGC1alpha pathway. *Journal of Pain Research*.

[150] Liu W., Lv Y., Ren F. (2018). PI3K/Akt pathway is required for spinal central sensitization in neuropathic pain. *Cellular and Molecular Neurobiology*.

[151] Martin L. J., Smith S. B., Khoutorsky A. (2017). Epiregulin and EGFR interactions are involved in pain processing. *The Journal of Clinical Investigation*.

[152] Scheff N. N., Ye Y., Conley Z. R. (2020). A disintegrin and metalloproteinase domain 17-epidermal growth factor receptor signaling contributes to oral cancer pain. *Pain*.

[153] Sun R., Yan J., Willis W. D. (2007). Activation of protein kinase B/Akt in the periphery contributes to pain behavior induced by capsaicin in rats. *Neuroscience*.

[154] Wang S., Liu S., Xu L. (2019). The upregulation of EGFR in the dorsal root ganglion contributes to chronic compression of dorsal root ganglions-induced neuropathic pain in rats. *Molecular Pain*.

[155] Yan Y., Liang Y., Ding T., Chu H. (2019). PI3K/Akt signaling pathway may be involved in MCP-1-induced P2X4R expression in cultured microglia and cancer-induced bone pain rats. *Neuroscience Letters*.

[156] Zhang W., Suo M., Yu G., Zhang M. (2019). Antinociceptive and anti-inflammatory effects of cryptotanshinone through PI3K/Akt signaling pathway in a rat model of neuropathic pain. *Chem Biol Interact*.

[157] Han M. M., Yang C. W., Cheung C. W., Li J. (2018). Blockage of spinal endothelin A receptors attenuates bone cancer pain via regulation of the Akt/ERK signaling pathway in mice. *Neuropeptides*.

[158] Rivera R. R., Lin M. E., Bornhop E. C., Chun J. (2020). Conditional Lpar1 gene targeting identifies cell types mediating neuropathic pain. *Federation of American Societies for Experimental Biology Journal*.

[159] Srikanth M., Chew W. S., Hind T. (2018). Lysophosphatidic acid and its receptor LPA1 mediate carrageenan induced inflammatory pain in mice. *European Journal of Pharmacology*.

[160] Uchida H., Nagai J., Ueda H. (2014). Lysophosphatidic acid and its receptors LPA1 and LPA3 mediate paclitaxel-induced neuropathic pain in mice. *Molecular Pain*.

[161] Ueda H., Neyama H. (2017). LPA1 receptor involvement in fibromyalgia-like pain induced by intermittent psychological stress, empathy. *Neurobiology of Pain*.

[162] Ueda H., Neyama H., Matsushita Y. (2020). Lysophosphatidic acid receptor 1- and 3-mediated hyperalgesia and hypoalgesia in diabetic neuropathic pain models in mice. *Cells*.

[163] Ueda H., Neyama H., Sasaki K., Miyama C., Iwamoto R. (2019). Lysophosphatidic acid LPA1 and LPA3 receptors play roles in the maintenance of late tissue plasminogen activator-induced central poststroke pain in mice. *Neurobiol Pain*.

[164] Wu J. X., Yuan X. M., Wang Q., Wei W., Xu M. Y. (2016). Rho/ROCK acts downstream of lysophosphatidic acid receptor 1 in modulating P2X3 receptor-mediated bone cancer pain in rats. *Molecular Pain*.

[165] Wu X. P., Yang Y. P., She R. X., Xing Z. M., Chen H. W., Zhang Y. W. (2019). microRNA-329 reduces bone cancer pain through the LPAR1-dependent LPAR1/ERK signal transduction pathway in mice. *Therapeutic Advances in Medical Oncology*.

[166] Ma L., Uchida H., Nagai J. (2009). Lysophosphatidic acid-3 receptor-mediated feed-forward production of lysophosphatidic acid: an initiator of nerve injury-induced neuropathic pain. *Molecular Pain*.

[167] Wei M., Li L., Zhang Y., Zhang M., Su Z. (2020). Downregulated circular RNA zRANB1 mediates Wnt5a/beta-Catenin signaling to promote neuropathic pain via miR-24-3p/LPAR3 axis in CCI rat models. *Gene*.

[168] Yue S. J., Liu J., Feng W. W. (2017). System pharmacology-based dissection of the synergistic mechanism of huangqi and huanglian for diabetes mellitus. *Frontiers in Pharmacology*.

[169] Chen C. M., Chen W. L., Hung C. T. (2019). Shaoyao Gancao Tang (SG-Tang), a formulated Chinese medicine, reduces aggregation and exerts neuroprotection in spinocerebellar ataxia type 17 (SCA17) cell and mouse models. *Aging (Albany NY)*.

[170] Jeong S. J., Lim H. S., Seo C. S. (2015). Traditional herbal formula Jakyakgamcho-tang (*Paeonia lactiflora* and *Glycyrrhiza uralensis*) impairs inflammatory chemokine production by inhibiting activation of STAT1 and NF-*κ*B in HaCaT cells. *Phytomedicine*.

[171] Kang T. H., Baek H. Y., Kim Y. C. (2005). Protective effect of jakyak-gamcho-tang extract and its constituents against t-BHP-induced oxidative damage in HT22 cells. *The American Journal of Chinese Medicine*.

[172] Maeda T., Shinozuka K., Baba K., Hayashi M., Hayashi E. (1983). Effect of shakuyaku-kanzoh-toh, a prescription composed of shakuyaku (Paeoniae Radix) and kanzoh (Glycyrrhizae Radix) on Guinea pig ileum. *J Pharmacobiodyn*.

[173] Cohen S. P., Mao J. (2014). Neuropathic pain: mechanisms and their clinical implications. *BMJ*.

[174] Gierthmuhlen J., Baron R. (2016). Neuropathic pain. *Seminars in Neurology*.

[175] Heinzmann S., McMahon S. B. (2011). New molecules for the treatment of pain. *Current Opinion in Supportive and Palliative Care*.

[176] Huang J., Zhang X., McNaughton P. A. (2006). Inflammatory pain: the cellular basis of heat hyperalgesia. *Current Neuropharmacology*.

[177] Inoue M., Rashid M. H., Fujita R., Contos J. J., Chun J., Ueda H. (2004). Initiation of neuropathic pain requires lysophosphatidic acid receptor signaling. *Nature Medicine*.

[178] Ji R. R., Strichartz G. (2004). Cell signaling and the genesis of neuropathic pain. *Science’s STKE*.

[179] Ji R. R., Xu Z. Z., Gao Y. J. (2014). Emerging targets in neuroinflammation-driven chronic pain. *Nature Reviews Drug Discovery*.

[180] Pezet S., Marchand F., D’Mello R. (2008). Phosphatidylinositol 3-kinase is a key mediator of central sensitization in painful inflammatory conditions. *The Journal of Neuroscience*.

[181] Price T. J., Basbaum A. I., Bresnahan J. (2018). Transition to chronic pain: opportunities for novel therapeutics. *Nature Reviews Neuroscience*.

[182] Raoof R., Willemen H., Eijkelkamp N. (2018). Divergent roles of immune cells and their mediators in pain. *Rheumatology (Oxford)*.

[183] Sah D. W., Ossipo M. H., Porreca F. (2003). Neurotrophic factors as novel therapeutics for neuropathic pain. *Nature Reviews Drug discovery*.

[184] Scanzello C. R. (2017). Chemokines and inflammation in osteoarthritis: insights from patients and animal models. *Journal of Orthopaedic Research*.

[185] Sisignano M., Baron R., Scholich K., Geisslinger G. (2014). Mechanism-based treatment for chemotherapy-induced peripheral neuropathic pain. *Nature Reviews Neurology*.

[186] White F. A., Bhangoo S. K., Miller R. J. (2005). Chemokines: integrators of pain and inflammation. *Nature Reviews Drug Discovery*.

[187] Calvo M., Zhu N., Tsantoulas C. (2010). Neuregulin-ErbB signaling promotes microglial proliferation and chemotaxis contributing to microgliosis and pain after peripheral nerve injury. *The Journal of Neuroscience*.

[188] Chen S. P., Zhou Y. Q., Liu D. Q. (2017). PI3K/Akt pathway: a potential therapeutic target for chronic pain. *Current Pharmaceutical Design*.

[189] Cui J., He W., Yi B. (2014). mTOR pathway is involved in ADP-evoked astrocyte activation and ATP release in the spinal dorsal horn in a rat neuropathic pain model. *Neuroscience*.

[190] Ding H. H., Zhang S. B., Lv Y. Y. (2019). TNF-alpha/STAT3 pathway epigenetically upregulates Nav1.6 expression in DRG and contributes to neuropathic pain induced by L5-VRT. *Journal of Neuroinflammation*.

[191] Fang D., Kong L. Y., Cai J. (2015). Interleukin-6-mediated functional upregulation of TRPV1 receptors in dorsal root ganglion neurons through the activation of JAK/PI3K signaling pathway: roles in the development of bone cancer pain in a rat model. *Pain*.

[192] Fang X., Zhou H., Huang S., Liu J. (2019). MiR-1906 attenuates neuropathic pain in rats by regulating the TLR4/mTOR/akt signaling pathway. *Translational Neuroscience*.

[193] Feng X. L., Deng H. B., Wang Z. G., Wu Y., Ke J. J., Feng X. B. (2019). Suberoylanilide hydroxamic acid triggers autophagy by influencing the mTOR pathway in the spinal dorsal horn in a rat neuropathic pain model. *Neurochemical Research*.

[194] Gu W., Sun Y., Gu W. (2020). The analgesic effects of pioglitazone in the bone cancer pain rats via regulating the PPAR*γ*/PTEN/mTOR signaling pathway in the spinal dorsal horn. *Biomedicine & Pharmacotherapy*.

[195] Gui Y., Li A., Chen F. (2015). Involvement of AMPK/SIRT1 pathway in anti-allodynic effect of troxerutin in CCI-induced neuropathic pain. *European Journal of Pharmacology*.

[196] Hao S., Mata M., Glorioso J. C., Fink D. J. (2007). Gene transfer to interfere with TNFalpha signaling in neuropathic pain. *Gene Therapy*.

[197] Huang J., Chen D., Yan F. (2020). JTC-801 alleviates mechanical allodynia in paclitaxel-induced neuropathic pain through the PI3K/Akt pathway. *European Journal of Pharmacology*.

[198] Jiang S. P., Zhang Z. D., Kang L. M., Wang Q. H., Zhang L., Chen H. P. (2016). Celecoxib reverts oxaliplatin-induced neuropathic pain through inhibiting PI3K/Akt2 pathway in the mouse dorsal root ganglion. *Experimental Neurology*.

[199] Kun L., Lu L., Yongda L., Xingyue L., Guang H. (2019). Hyperbaric oxygen promotes mitophagy by activating CaMKK*β*/AMPK signal pathway in rats of neuropathic pain. *Molecular Pain*.

[200] Lee G. W., Son J. Y., Lee A. R., Ju J. S., Bae Y. C., Ahn D. K. (2019). Central VEGF-A pathway plays a key role in the development of trigeminal neuropathic pain in rats. *Molecular Pain*.

[201] Liu Y. D., Wang Z. B., Han G., Jin L., Zhao P. (2019). Hyperbaric oxygen relieves neuropathic pain through AKT/TSC2/mTOR pathway activity to induce autophagy. *Journal of Pain Research*.

[202] Liu Y. D., Wang Z. B., Han G., Zhao P. (2017). Hyperbaric oxygen treatment attenuates neuropathic pain by elevating autophagy flux via inhibiting mTOR pathway. *American Journal of Translational Research*.

[203] Lu Y., Jiang B. C., Cao D. L. (2014). TRAF6 upregulation in spinal astrocytes maintains neuropathic pain by integrating TNF-alpha and IL-1*β* signaling. *Pain*.

[204] Maixner D. W., Yan X., Gao M., Yadav R., Weng H. R. (2015). Adenosine monophosphate-activated protein kinase regulates interleukin-1*β* expression and glial glutamate transporter function in rodents with neuropathic pain. *Anesthesiology*.

[205] Mejia G. L., Asiedu M. N., Hitoshi Y., Dussor G., Price T. J. (2016). The potent, indirect adenosine monophosphate- activated protein kinase activator R419 attenuates mitogen-activated protein kinase signaling, inhibits nociceptor excitability, and reduces pain hypersensitivity in mice. *Pain Reports*.

[206] Obara I., Tochiki K. K., Geranton S. M. (2011). Systemic inhibition of the mammalian target of rapamycin (mTOR) pathway reduces neuropathic pain in mice. *Pain*.

[207] Price T. J., Das V., Dussor G. (2016). Adenosine monophosphate-activated protein kinase (AMPK) activators for the prevention, treatment and potential reversal of pathological pain. *Current Drug Targets*.

[208] Sabsovich I., Guo T. Z., Wei T. (2008). TNF signaling contributes to the development of nociceptive sensitization in a tibia fracture model of complex regional pain syndrome type I. *Pain*.

[209] Shi J., Jiang K., Li Z. (2018). MiR-145 ameliorates neuropathic pain via inhibiting inflammatory responses and mTOR signaling pathway by targeting Akt3 in a rat model. *Neuroscience Research*.

[210] Sobeh M., Mahmoud M. F., Rezq S. (2020). *Haematoxylon campechianum* extract ameliorates neuropathic pain via inhibition of NF-*κ*B/TNF-*α*/NOX/iNOS signalling pathway in a rat model of chronic constriction injury. *Biomolecules*.

[211] Sobeh M., Mahmoud M. F., Rezq S. (2019). *Salix tetrasperma* roxb. Extract alleviates neuropathic pain in rats via modulation of the NF-*κ*B/TNF-*α*/NOX/iNOS pathway. *Antioxidants (Basel)*.

[212] Song H., Han Y., Pan C. (2015). Activation of adenosine monophosphate-activated protein kinase suppresses neuroinflammation and ameliorates bone cancer pain: involvement of inhibition on mitogen-activated protein kinase. *Anesthesiology*.

[213] Tao F., Li Q., Liu S. (2013). Role of neuregulin-1/ErbB signaling in stem cell therapy for spinal cord injury-induced chronic neuropathic pain. *Stem Cells*.

[214] Wang R., Qiu Z., Wang G. (2020). Quercetin attenuates diabetic neuropathic pain by inhibiting mTOR/p70S6K pathway-mediated changes of synaptic morphology and synaptic protein levels in spinal dorsal horn of db/db mice. *European Journal of Pharmacology*.

[215] Xie X., Ma L., Xi K., Zhang W., Fan D. (2017). MicroRNA-183 suppresses neuropathic pain and expression of AMPA receptors by targeting mTOR/VEGF signaling pathway. *Cell Physiol Biochem*.

[216] Xing X., Wu K., Dong Y. (2020). Hyperactive Akt-mTOR pathway as a therapeutic target for pain hypersensitivity in Cntnap2-deficient mice. *Neuropharmacology*.

[217] Yang G., Tan Q., Li Z. (2020). The AMPK pathway triggers autophagy during CSF1-induced microglial activation and may be implicated in inducing neuropathic pain. *Journal of Neuroimmunology*.

[218] Yang Q. Q., Li H. N., Zhang S. T. (2020). Red nucleus IL-6 mediates the maintenance of neuropathic pain by inducing the productions of TNF-*α* and IL-1*β* through the JAK2/STAT3 and ERK signaling pathways. *Neuropathology*.

[219] Yuan L., Liu C., Wan Y., Yan H., Li T. (2019). Effect of HDAC2/Inpp5f on neuropathic pain and cognitive function through regulating PI3K/Akt/GSK-3*β* signal pathway in rats with neuropathic pain. *Experimental and Therapeutic Medicine*.

[220] Zhang J., Wang L., Wang H., Su Z., Pang X. (2019). Neuroinflammation and central PI3K/Akt/mTOR signal pathway contribute to bone cancer pain. *Molecular Pain*.

[221] Zhang Q., Yu J., Wang J. (2015). The red nucleus TNF-alpha participates in the initiation and maintenance of neuropathic pain through different signaling pathways. *Neurochemical Research*.

[222] Zhang W., Bai Y., Qiao Y. (2018). 8-O-acetyl shanzhiside methylester from lamiophlomis rotata reduces neuropathic pain by inhibiting the ERK/TNF-alpha pathway in spinal astrocytes. *Frontiers in Cellular Neuroscience*.

[223] Zhang Y., Yuan L., Chen Y., Lin C., Ye G. (2019). Oxyntomodulin attenuates TNF*α* induced neuropathic pain by inhibiting the activation of the NF*κ*B pathway. *Molecular Medicine Reports*.

[224] Zhang Z., Deng M., Huang J. (2020). Microglial annexin A3 downregulation alleviates bone cancer-induced pain via inhibiting the Hif-1alpha/VEGF signaling pathway. *Pain*.

[225] Zhao D., Han D. F., Wang S. S., Lv B., Wang X., Ma C. (2019). Roles of tumor necrosis factor-alpha and interleukin-6 in regulating bone cancer pain via TRPA1 signal pathway and beneficial effects of inhibition of neuro-inflammation and TRPA1. *Molecular Pain*.

[226] Ji A., Zhu M. (2020). Effects of curcumin on biological behavior and NF-*κ*B/TNF-*α* pathway in mice with metastatic bone pain of breast cancer induced by walker 256 cells. *J Cancer Ther*.

[227] Dehghani S., Alipoor E., Salimzadeh A. (2018). The effect of a garlic supplement on the pro-inflammatory adipocytokines, resistin and tumor necrosis factor-alpha, and on pain severity, in overweight or obese women with knee osteoarthritis. *Phytomedicine*.

[228] Tian Y., Wang S., Ma Y., Lim G., Kim H., Mao J. (2011). Leptin enhances NMDA-induced spinal excitation in rats: a functional link between adipocytokine and neuropathic pain. *Pain*.

[229] Ji R., Zhuang Z., Xu H., Clapham D. J. T. J. o.P. (2005). Phosphatidylinositol 3-kinase (PI3K) cascade is a novel signaling pathway to mediate inflammatory pain. *Journal of Pain*.

[230] Ji Y., Tang B., Traub R. J. (2011). Spinal estrogen receptor alpha mediates estradiol-induced pronociception in a visceral pain model in the rat. *Pain*.

[231] Khariv V., Acioglu C., Ni L. (2018). A link between plasma membrane calcium ATPase 2 (PMCA2), estrogen and estrogen receptor alpha signaling in mechanical pain. *Scientific Reports*.

[232] Piu F., Cheevers C., Hyldtoft L. (2008). Broad modulation of neuropathic pain states by a selective estrogen receptor beta agonist. *European Journal of Pharmacology*.

[233] Tang B., Ji Y., Traub R. J. (2008). Estrogen alters spinal NMDA receptor activity via a PKA signaling pathway in a visceral pain model in the rat. *Pain*.

[234] Yan X. J., Feng C. C., Liu Q. (2014). Vagal afferents mediate antinociception of estrogen in a rat model of visceral pain: the involvement of intestinal mucosal mast cells and 5-hydroxytryptamine 3 signaling. *The Journal of Pain*.

[235] Zielinska M., Fichna J., Bashashati M. (2017). G protein-coupled estrogen receptor and estrogen receptor ligands regulate colonic motility and visceral pain. *Neurogastroenterology & Motility*.

[236] Busch-Dienstfertig M., Gonzalez-Rodriguez S. (2013). IL-4, JAK-STAT signaling, and pain. *JAKSTAT*.

[237] Cao S., Li Y., Wang L. (2016). Synergistic analgesic effect of propofol-alfentanil combination through detecting the inhibition of cAMP signal pathway. *Journal of Pharmacy and Pharmacology*.

[238] Chen S. P., Sun J., Zhou Y. Q. (2018). Sinomenine attenuates cancer-induced bone pain via suppressing microglial JAK2/STAT3 and neuronal CAMKII/CREB cascades in rat models. *Molecular Pain*.

[239] Hang L. H., Yang J. P., Shao D. H., Chen Z., Wang H. (2013). Involvement of spinal PKA/CREB signaling pathway in the development of bone cancer pain. *Pharmacological Reports*.

[240] Hsiao H. T., Lin Y. C., Wang J. C., Tsai Y. C., Liu Y. C. (2016). Hypoxia inducible factor-1*α* inhibition produced anti-allodynia effect and suppressed inflammatory cytokine production in early stage of mouse complex regional pain syndrome model. *Clinical and Experimental Pharmacology and Physiology*.

[241] Hsiao H. T., Liu Y. Y., Wang J. C., Lin Y. C., Liu Y. C. (2019). The analgesic effect of propofol associated with the inhibition of hypoxia inducible factor and inflammasome in complex regional pain syndrome. *Journal of Biomedical Science*.

[242] Hsieh Y. L., Chou L. W., Chang P. L., Yang C. C., Kao M. J., Hong C. Z. (2012). Low-level laser therapy alleviates neuropathic pain and promotes function recovery in rats with chronic constriction injury: possible involvements in hypoxia-inducible factor 1alpha (HIF-1*α*). *The Journal of Comparative Neurology*.

[243] Kallenborn-Gerhardt W., Metzner K., Lu R. (2020). Neuropathic and cAMP-induced pain behavior is ameliorated in mice lacking CNGB1. *Neuropharmacology*.

[244] Kober K. M., Lee M. C., Olshen A. (2020). Differential methylation and expression of genes in the hypoxia-inducible factor 1 signaling pathway are associated with paclitaxel-induced peripheral neuropathy in breast cancer survivors and with preclinical models of chemotherapy-induced neuropathic pain. *Molecular Pain*.

[245] Liou J. T., Liu F. C., Hsin S. T., Yang C. Y., Lui P. W. (2007). Inhibition of the cyclic adenosine monophosphate pathway attenuates neuropathic pain and reduces phosphorylation of cyclic adenosine monophosphate response element-binding in the spinal cord after partial sciatic nerve ligation in rats. *Anesthesia & Analgesia*.

[246] Ludman T., Melemedjian O. K. (2019). Bortezomib and metformin opposingly regulate the expression of hypoxia-inducible factor alpha and the consequent development of chemotherapy-induced painful peripheral neuropathy. *Molecular Pain*.

[247] Rojas D. R., Tegeder I., Kuner R., Agarwal N. (2018). Hypoxia-inducible factor 1alpha protects peripheral sensory neurons from diabetic peripheral neuropathy by suppressing accumulation of reactive oxygen species. *Journal of Molecular Medicine*.

[248] Salaffi F., Giacobazzi G., Di Carlo M. (2018). Chronic pain in inflammatory arthritis: mechanisms, metrology, and emerging targets-A focus on the JAK-STAT pathway. *Pain Research and Management*.

[249] Salehi F., Hosseini-Zare M. S., Aghajani H., Seyedi S. Y., Hosseini-Zare M. S., Sharifzadeh M. (2017). Effect of bucladesine, pentoxifylline, and H-89 as cyclic adenosine monophosphate analog, phosphodiesterase, and protein kinase A inhibitor on acute pain. *Fundamental & Clinical Pharmacology*.

[250] Shao X. M., Sun J., Jiang Y. L. (2016). Inhibition of the cAMP/PKA/CREB pathway contributes to the analgesic effects of electroacupuncture in the anterior cingulate cortex in a rat pain memory model. *Neural Plasticity*.

[251] Tang J., Li Z. H., Ge S. N. (2012). The inhibition of spinal astrocytic JAK2-STAT3 pathway activation correlates with the analgesic effects of triptolide in the rat neuropathic pain model. *Evidence-Based Complementary and Alternative Medicine*.

[252] Wang H., Huo X., Chen H. (2018). Hydrogen-rich saline activated autophagy via HIF-1alpha pathways in neuropathic pain model. *BioMed Research International*.

[253] Xiong Z., Ding J., Zhou J., Yao S., Zheng J., Guo X. (2020). Correlation between the HIF-1*α*/Notch signaling pathway and Modic changes in nucleus pulposus cells isolated from patients with low back pain. *BMC Musculoskeletal Disorders*.

[254] Xun S., Zheng R. (2020). Dexmedetomidine alleviates neuropathic pain by regulating JAK/STAT pathway in rats. *Journal of Cellular Biochemistry*.

[255] Zhang G., Feng G. Y., Guo Y. R., Liang D. Q., Yuan Y., Wang H. L. (2017). Correlation between liver cancer pain and the HIF-1 and VEGF expression levels. *Oncology Letters*.

[256] Zhu G. Q., Liu S., He D. D., Liu Y. P., Song X. J. (2014). Activation of the cAMP-PKA signaling pathway in rat dorsal root ganglion and spinal cord contributes toward induction and maintenance of bone cancer pain. *Behavioural Pharmacology*.

[257] Skyba D. A., Radhakrishnan R., Bement M. K. H., Sluka K. A. (2004). The cAMP pathway and pain: potential targets for drug development. *Drug Discovery Today: Disease Models*.

[258] Androutsopoulos V. P., Ruparelia K., Arroo R. R., Tsatsakis A. M., Spandidos D. A. (2009). CYP1-mediated antiproliferative activity of dietary flavonoids in MDA-MB-468 breast cancer cells. *Toxicology*.

[259] Chao M. V., Rajagopal R., Lee F. S. (2006). Neurotrophin signalling in health and disease. *Clinical Science (London)*.

[260] Hu X. M., Zhang H., Xu H. (2017). Chemokine receptor CXCR4 regulates CaMKII/CREB pathway in spinal neurons that underlies cancer-induced bone pain. *Scientific Reports*.

[261] Khan N., Smith M. T. (2015). Neurotrophins and neuropathic pain: role in pathobiology. *Molecules*.

[262] Mannion R. J., Costigan M., Decosterd I. (1999). Neurotrophins: peripherally and centrally acting modulators of tactile stimulus-induced inflammatory pain hypersensitivity. *Proceedings of the National Academy of Sciences of the United States of America*.

[263] Pezet S., McMahon S. B. (2006). Neurotrophins: mediators and modulators of pain. *Annual Review of Neuroscience*.

[264] Xu H., Peng C., Chen X. T. (2020). Chemokine receptor CXCR4 activates the RhoA/ROCK2 pathway in spinal neurons that induces bone cancer pain. *Molecular Pain*.

[265] Zhang Q., Cao D. L., Zhang Z. J., Jiang B. C., Gao Y. J. (2016). Chemokine CXCL13 mediates orofacial neuropathic pain via CXCR5/ERK pathway in the trigeminal ganglion of mice. *Journal of Neuroinflammation*.

[266] Brussee V., Cunningham F. A., Zochodne D. W. (2004). Direct insulin signaling of neurons reverses diabetic neuropathy. *Diabetes*.

[267] Dobretsov M., Ghaleb A. H., Romanovsky D., Pablo C. S., Stimers J. R. (2007). Impaired insulin signaling as a potential trigger of pain in diabetes and prediabetes. *International Anesthesiology Clinics*.

[268] Sharma S., Chopra K., Kulkarni S. K. (2007). Effect of insulin and its combination with resveratrol or curcumin in attenuation of diabetic neuropathic pain: participation of nitric oxide and TNF-alpha. *Phytotherapy Research*.

[269] Sugimoto K., Rashid I. B., Shoji M., Suda T., Yasujima M. (2008). Early changes in insulin receptor signaling and pain sensation in streptozotocin-induced diabetic neuropathy in rats. *The Journal of Pain*.

[270] Banimostafavi E. S., Fakhar M., Abediankenari S. (2020). Determining serum levels of IL-10 and IL-17 in patients with low back pain caused by lumbar disc degeneration. *Infectious Disorders Drug Targets*.

[271] Day Y. J., Liou J. T., Lee C. M. (2014). Lack of interleukin-17 leads to a modulated micro-environment and amelioration of mechanical hypersensitivity after peripheral nerve injury in mice. *Pain*.

[272] Hu X., Huang F., Wang Z. J. (2018). CaMKIIalpha mediates the effect of IL-17 to promote ongoing spontaneous and evoked pain in multiple sclerosis. *The Journal of Neuroscience*.

[273] Kim C. F., Moalem-Taylor G. (2011). Interleukin-17 contributes to neuroinflammation and neuropathic pain following peripheral nerve injury in mice. *The Journal of Pain*.

[274] Liu X., Fan S., Zheng M., Chen J., Zhang J., Li H. (2017). The mediation of interleukin-17 and chemokine ligand 2 in pelvic pain of experimental autoimmune prostatitis. *Experimental and Therapeutic Medicine*.

[275] Luo H., Liu H. Z., Zhang W. W. (2019). Interleukin-17 regulates neuron-glial communications, synaptic transmission, and neuropathic pain after chemotherapy. *Cell Reports*.

[276] Meng X., Zhang Y., Lao L. (2013). Spinal interleukin-17 promotes thermal hyperalgesia and NMDA NR1 phosphorylation in an inflammatory pain rat model. *Pain*.

[277] Noma N., Khan J., Chen I. F. (2011). Interleukin-17 levels in rat models of nerve damage and neuropathic pain. *Neuroscience Letters*.

[278] Richter F., Natura G., Ebbinghaus M. (2012). Interleukin-17 sensitizes joint nociceptors to mechanical stimuli and contributes to arthritic pain through neuronal interleukin-17 receptors in rodents. *Arthritis & Rheumatology*.

[279] Sun C., Zhang J., Chen L. (2017). IL-17 contributed to the neuropathic pain following peripheral nerve injury by promoting astrocyte proliferation and secretion of proinflammatory cytokines. *Molecular Medicine Reports*.

[280] Yao C. Y., Weng Z. L., Zhang J. C., Feng T., Lin Y., Yao S. (2016). Interleukin-17A acts to maintain neuropathic pain through activation of CaMKII/CREB signaling in spinal neurons. *Molecular Neurobiology*.

[281] Zhang W., Nie L., Guo Y. J. (2014). Th17 cell frequency and IL-17 concentration correlate with pre- and postoperative pain sensation in patients with intervertebral disk degeneration. *Orthopedics*.

[282] Zou D., Zhang K., Yang Y. (2018). Th17 and IL-17 exhibit higher levels in osteonecrosis of the femoral head and have a positive correlation with severity of pain. *Endokrynologia Polska*.

[283] Bieglmayer C., Hofer G., Kainz C., Reinthaller A., Kopp B., Janisch H. (1995). Concentrations of various arachidonic acid metabolites in menstrual fluid are associated with menstrual pain and are influenced by hormonal contraceptives. *Gynecological Endocrinology*.

[284] Sinning C., Watzer B., De Petrocellis L., Di Marzo V., Imming P. (2008). Dopamides, vanillylamides, ethanolamides, and arachidonic acid amides of anti-inflammatory and analgesic drug substances as TRPV1 ligands. *ChemMedChem*.

[285] Smith H. S. (2006). Arachidonic acid pathways in nociception. *The Journal of Supportive Oncology*.

[286] Sung B., Wang S., Zhou B. (2007). Altered spinal arachidonic acid turnover after peripheral nerve injury regulates regional glutamate concentration and neuropathic pain behaviors in rats. *Pain*.

[287] Yang W., Yaggie R. E., Schaeffer A. J., Klumpp D. J. (2020). AOAH remodels arachidonic acid-containing phospholipid pools in a model of interstitial cystitis pain: a MAPP network study. *PLoS One*.

[288] Wang X., Wu T., Lee Y., Dionne R. J. C. P., Therapeutics (2005). Gene expression in the arachidonic acid pathway due to tissue injury and inflammation, an NSAID, and COXIB in a clinical model of pain. *Clinical Pharmacology & Therapeutics*.

[289] Haleem D. J., Nawaz S., Salman T. (2018). Dopamine and serotonin metabolism associated with morphine reward and its inhibition with buspirone: a study in the rat striatum. *Pharmacology Biochemistry and Behavior*.

[290] Li N., Li C., Han R. (2019). LPM580098, a novel triple reuptake inhibitor of serotonin, noradrenaline, and dopamine, attenuates neuropathic pain. *Frontiers in Pharmacology*.

[291] Martikainen I. K., Hagelberg N., Jaaskelainen S. K., Hietala J., Pertovaara A. (2018). Dopaminergic and serotonergic mechanisms in the modulation of pain: in vivo studies in human brain. *European Journal of Pharmacology*.

[292] Sagheddu C., Aroni S., De Felice M. (2015). Enhanced serotonin and mesolimbic dopamine transmissions in a rat model of neuropathic pain. *Neuropharmacology*.

[293] Li L., Qiu H., Liu M., Cai Y. (2020). A network pharmacology-based study of the molecular mechanisms of shaoyao-gancao decoction in treating Parkinson’s disease. *Interdisciplinary Sciences*.

[294] Zhu N., Hou J., Ma G., Liu J. (2019). Network pharmacology identifies the mechanisms of action of shaoyao gancao decoction in the treatment of osteoarthritis. *Medical Science Monitor*.

[295] Addepalli V., Suryavanshi S. V. (2018). Catechin attenuates diabetic autonomic neuropathy in streptozotocin induced diabetic rats. *Biomedicine & Pharmacotherapy*.

[296] Deciga-Campos M., Mata R., Rivero-Cruz I. (2017). Antinociceptive pharmacological profile of dysphania graveolens in mouse. *Biomedicine & Pharmacotherapy*.

[297] Islam S., Shajib M. S., Rashid R. B. (2019). Antinociceptive activities of Artocarpus lacucha Buch-ham (Moraceae) and its isolated phenolic compound, catechin, in mice. *BMC Complementary and Alternative Medicine*.

[298] Zhang Y., Sun D., Meng Q., Guo W., Chen Q., Zhang Y. (2016). Calcium channels contribute to albiflorin-mediated antinociceptive effects in mouse model. *Neuroscience Letters*.

[299] Lima Cavendish R., de Souza Santos J., Belo Neto R. (2015). Antinociceptive and anti-inflammatory effects of Brazilian red propolis extract and formononetin in rodents. *Journal of Ethnopharmacology*.

[300] Parlar A., Arslan S. O., Cam S. A. (2020). Glabridin alleviates inflammation and nociception in rodents by activating BKCa channels and reducing NO levels. *Biological and Pharmaceutical Bulletin*.

[301] Wang H. L., Li Y. X., Niu Y. T. (2015). Observing anti-inflammatory and anti-nociceptive activities of glycyrrhizin through regulating COX-2 and pro-inflammatory cytokines expressions in mice. *Inflammation*.

[302] Wang X. S., Guan S. Y., Liu A. (2019). Anxiolytic effects of formononetin in an inflammatory pain mouse model. *Mol Brain*.

[303] Farbood Y., Sarkaki A., Hashemi S., Mansouri M. T., Dianat M. (2013). The effects of gallic acid on pain and memory following transient global ischemia/reperfusion in Wistar rats. *Avicenna Journal of Phytomedicine*.

[304] Hajimoradi M., Fazilati M., Gharib-Naseri M. K., Sarkaki A. (2015). Gallic acid and exercise training improve motor function, nerve conduction velocity but not pain sense reflex after experimental sciatic nerve crush in male rats. *Avicenna Journal of Phytomedicine*.

[305] Feldman P., Due M. R., Ripsch M. S., Khanna R., White F. A. (2012). The persistent release of HMGB1 contributes to tactile hyperalgesia in a rodent model of neuropathic pain. *Journal of Neuroinflammation*.

[306] Hu C. Y., Zhao Y. T. (2014). Analgesic effects of naringenin in rats with spinal nerve ligation-induced neuropathic pain. *Biomedical Reports*.

[307] Manchope M. F., Calixto-Campos C., Coelho-Silva L. (2016). Naringenin inhibits superoxide anion-induced inflammatory pain: role of oxidative stress, cytokines, nrf-2 and the NO-cGMP-PKG-KATP channel signaling pathway. *PLoS One*.

[308] Manchope M. F., Casagrande R., Verri W. A. (2017). Naringenin: an analgesic and anti-inflammatory citrus flavanone. *Oncotarget*.

[309] Pinho-Ribeiro F. A., Zarpelon A. C., Fattori V. (2016). Naringenin reduces inflammatory pain in mice. *Neuropharmacology*.

[310] Pinho-Ribeiro F. A., Zarpelon A. C., Mizokami S. S. (2016). The citrus flavonone naringenin reduces lipopolysaccharide-induced inflammatory pain and leukocyte recruitment by inhibiting NF-*κ*B activation. *The Journal of Nutritional Biochemistry*.

[311] Singh P., Bansal S., Kuhad A., Kumar A., Chopra K. (2020). Naringenin ameliorates diabetic neuropathic pain by modulation of oxidative-nitrosative stress, cytokines and MMP-9 levels. *Food & Function*.

[312] Xue N., Wu X., Wu L., Li L., Wang F. (2019). Antinociceptive and anti-inflammatory effect of naringenin in different nociceptive and inflammatory mice models. *Life Sciences*.

[313] Ruiz-Miyazawa K. W., Borghi S. M., Pinho-Ribeiro F. A. (2018). The citrus flavanone naringenin reduces gout-induced joint pain and inflammation in mice by inhibiting the activation of NF*κ*B and macrophage release of IL-1*β*. *Journal of Functional Foods*.

[314] Dallazen J. L., da Silva C. F., Hamm L. (2017). Further antinociceptive properties of naringenin on acute and chronic pain in mice. *Natural Product Communications*.

[315] Sato Y., He J. X., Nagai H., Tani T., Akao T. (2007). Isoliquiritigenin, one of the antispasmodic principles of *Glycyrrhiza ularensis* roots, acts in the lower part of intestine. *Biological and Pharmaceutical Bulletin*.

[316] Shi Y., Wu D., Sun Z. (2012). Analgesic and uterine relaxant effects of isoliquiritigenin, a flavone from *Glycyrrhiza glabra*. *Phytotherapy Research*.

[317] Jamali-Raeufy N., Baluchnejadmojarad T., Roghani M., Keimasi S., Goudarzi M. (2019). Isorhamnetin exerts neuroprotective effects in STZ-induced diabetic rats via attenuation of oxidative stress, inflammation and apoptosis. *Journal of Chemical Neuroanatomy*.

[318] Kim S. H., Park J. G., Sung G. H. (2015). Kaempferol, a dietary flavonoid, ameliorates acute inflammatory and nociceptive symptoms in gastritis, pancreatitis, and abdominal pain. *Molecular Nutrition & Food Research*.

[319] Kishore L., Kaur N., Singh R. (2018). Effect of kaempferol isolated from seeds of *Eruca sativa* on changes of pain sensitivity in streptozotocin-induced diabetic neuropathy. *Inflammopharmacology*.

[320] Parveen Z., Deng Y., Saeed M. K., Dai R., Ahamad W., Yu Y. H. (2007). Antiinflammatory and analgesic activities of Thesium chinense Turcz extracts and its major flavonoids, kaempferol and kaempferol-3-O-glucoside. *Yakugaku Zasshi*.

[321] Ahangarpour A., Oroojan A. A., Khorsandi L., Shabani R., Mojaddami S. (2016). Preventive effects of betulinic acid on streptozotocinnicotinamide induced diabetic nephropathy in male mouse. *Journal of Nephropathology*.

[322] Bellampalli S. S., Ji Y., Moutal A. (2019). Betulinic acid, derived from the desert lavender *Hyptis emoryi*, attenuates paclitaxel-, HIV-, and nerve injury-associated peripheral sensory neuropathy via block of N- and T-type calcium channels. *Pain*.

[323] Kalra J., Lingaraju M. C., Mathesh K. (2018). Betulinic acid alleviates dextran sulfate sodium-induced colitis and visceral pain in mice. *Naunyn-Schmiedeberg’s Archives of Pharmacology*.

[324] Oyebanji B. O., Saba A. B., Oridupa O. A. (2014). Studies on the anti-inflammatory, analgesic and antipyrexic activities of betulinic acid derived from *Tetracera potatoria*. *African journal of Traditional, Complementary, and Alternative Medicines*.

[325] Rios J. L., Manez S. (2018). New pharmacological opportunities for betulinic acid. *Planta Medica*.

[326] Yogeeswari P., Sriram D. (2005). Betulinic acid and its derivatives: a review on their biological properties. *Current Medicinal Chemistry*.

[327] Borghi S. M., Pinho-Ribeiro F. A., Fattori V. (2016). Quercetin inhibits peripheral and spinal cord nociceptive mechanisms to reduce intense acute swimming-induced muscle pain in mice. *PLoS One*.

[328] Calixto-Campos C., Correa M. P., Carvalho T. T. (2015). Quercetin reduces Ehrlich tumor-induced cancer pain in mice. *Analytical Cellular Pathology (Amsterdam)*.

[329] Carullo G., Cappello A. R., Frattaruolo L., Badolato M., Armentano B., Aiello F. (2017). Quercetin and derivatives: useful tools in inflammation and pain management. *Future Medicinal Chemistry*.

[330] Filho A. W., Filho V. C., Olinger L., de Souza M. M. (2008). Quercetin: further investigation of its antinociceptive properties and mechanisms of action. *Archives of Pharmacal Research*.

[331] Li Z., Zhang J., Ren X., Liu Q., Yang X. (2018). The mechanism of quercetin in regulating osteoclast activation and the PAR2/TRPV1 signaling pathway in the treatment of bone cancer pain. *International Journal of Clinical and Experimental Pathology*.

[332] Martinez A. L., Gonzalez-Trujano M. E., Aguirre-Hernandez E., Moreno J., Soto-Hernandez M., Lopez-Munoz F. J. (2009). Antinociceptive activity of *Tilia americana* var. Mexicana inflorescences and quercetin in the formalin test and in an arthritic pain model in rats. *Neuropharmacology*.

[333] Shoskes D. A., Nickel J. C. (2011). Quercetin for chronic prostatitis/chronic pelvic pain syndrome. *Urologic Clinics of North America*.

[334] Valerio D. A., Georgetti S. R., Magro D. A. (2009). Quercetin reduces inflammatory pain: inhibition of oxidative stress and cytokine production. *Journal of Natural Products*.

[335] Anjaneyulu M., Chopra K. (2003). Quercetin, a bioflavonoid, attenuates thermal hyperalgesia in a mouse model of diabetic neuropathic pain. *Prog Neuropsychopharmacol Biol Psychiatry*.

[336] Anjaneyulu M., Chopra K. (2004). Quercetin attenuates thermal hyperalgesia and cold allodynia in STZ-induced diabetic rats. *Indian Journal of Experimental Biology*.

[337] Azevedo M. I., Pereira A. F., Nogueira R. B. (2013). The antioxidant effects of the flavonoids rutin and quercetin inhibit oxaliplatin-induced chronic painful peripheral neuropathy. *Molecular Pain*.

[338] Dureshahwar K., Mubashir M., Une H. D. (2017). Quantification of quercetin obtained from *Allium cepa* Lam. leaves and its effects on streptozotocin-induced diabetic neuropathy. *Pharmacognosy Research*.

[339] Ferreira P. E., Lopes C. R., Alves A. M. (2013). Diabetic neuropathy: an evaluation of the use of quercetin in the cecum of rats. *World Journal of Gastroenterology*.

[340] Gao W., Zan Y., Wang Z. J., Hu X. Y., Huang F. (2016). Quercetin ameliorates paclitaxel-induced neuropathic pain by stabilizing mast cells, and subsequently blocking PKC*ε*-dependent activation of TRPV1. *Acta Pharmacologica Sinica*.

[341] Ji C., Xu Y., Han F. (2017). Quercetin alleviates thermal and cold hyperalgesia in a rat neuropathic pain model by inhibiting Toll-like receptor signaling. *Biomedicine & Pharmacotherapy*.

[342] Muto N., Matsuoka Y., Arakawa K. (2018). Quercetin attenuates neuropathic pain in rats with spared nerve injury. *Acta Medica Okayama*.

[343] Naidu P. S., Singh A., Kulkarni S. K. (2003). D2-dopamine receptor and alpha2-adrenoreceptor-mediated analgesic response of quercetin. *Indian Journal of Experimental Biology*.

[344] Raygude K. S., Kandhare A. D., Ghosh P., Ghule A. E., Bodhankar S. L. (2012). Evaluation of ameliorative effect of quercetin in experimental model of alcoholic neuropathy in rats. *Inflammopharmacology*.

[345] Xie J., Song W., Liang X. (2020). Protective effect of quercetin on streptozotocin-induced diabetic peripheral neuropathy rats through modulating gut microbiota and reactive oxygen species level. *Biomedicine & Pharmacotherapy*.

[346] Yang R., Li L., Yuan H. (2019). Quercetin relieved diabetic neuropathic pain by inhibiting upregulated P2X4 receptor in dorsal root ganglia. *Journal of Cellular Physiology*.

[347] Kandhare A. D., Raygude K. S., Kumar V. S. (2012). Ameliorative effects quercetin against impaired motor nerve function, inflammatory mediators and apoptosis in neonatal streptozotocin-induced diabetic neuropathy in rats. *Biomedicine and Aging Pathology*.

[348] Acikara O. B., Citoglu G. S., Dall’Acqua S. (2014). Bioassay-guided isolation of the antinociceptive compounds motiol and beta-sitosterol from *Scorzonera latifolia* root extract. *Pharmazie*.

[349] Das N., Bhattacharya A., Kumar Mandal S. (2018). *Ichnocarpus frutescens* (L.) R. Br. root derived phyto-steroids defends inflammation and algesia by pulling down the pro-inflammatory and nociceptive pain mediators: an in-vitro and in-vivo appraisal. *Steroids*.

[350] Hernandez-Flores M. E., Torres-Valencia J. M., Carino-Cortes R. (2019). In search of safe pain relief: the analgesic and anti-inflammatory activity of phytosteryl ibuprofenates. *Steroids*.

[351] Nirmal S. A., Pal S. C., Mandal S. C., Patil A. N. (2012). Analgesic and anti-inflammatory activity of beta-sitosterol isolated from *Nyctanthes arbortristis* leaves. *Inflammopharmacology*.

[352] Paniagua-Perez R., Flores-Mondragon G., Reyes-Legorreta C. (2017). Evaluation of the anti-inflammatory capacity of beta-sitosterol in rodent assays. *African Journal of Traditional, Complementary, and Alternative Medicines*.

[353] Santos A. R., Niero R., Filho V. C. (1995). Antinociceptive properties of steroids isolated from *Phyllanthus corcovadensis* in mice. *Planta Medica*.

[354] Battisti W. P., Katz N. P., Weaver A. L. (2004). Pain management in osteoarthritis: a focus on onset of efficacy—a comparison of rofecoxib, celecoxib, acetaminophen, and nabumetone across four clinical trials. *Journal of Pain*.

