# Supplementary material for: An Investigation of the Molecular Mechanisms Underlying the Analgesic Effect of Jakyak-Gamcho Decoction: A Network Pharmacology Study
Source: Evid Based Complement Alternat Med. 2020 Dec 1;2020:6628641. doi: 10.1155/2020/6628641 (PMC7732394; doi:10.1155/2020/6628641)
Supplement: Supplementary Materials — Supplementary Figure S1: contribution index analysis for the analgesic activity of active chemical compounds of Jakyak-Gamcho decoction. Supplementary Figure S2: functional enrichment analyses for the pain-associated targets of Jakyak-Gamcho decoction. Supplementary Figure S3: functional interaction analysis of the pain-associated targets of Jakyak-Gamcho decoction. Supplementary Table S1: list of the chemical compounds contained in Jakyak-Gamcho decoction. Supplementary Table S2: list of the active chemical compounds contained in Jakyak-Gamcho decoction. Supplementary Table S3: list of the targets of active chemical compounds of Jakyak-Gamcho decoction. Supplementary Table S4: docking scores of the active chemical compounds of Jakyak-Gamcho decoction with the hub targets. [file 6628641.f1.docx]

**Supplementary Materials**

**An investigation of the molecular mechanisms underlying the analgesic effect of Jakyak-Gamcho decoction: A network pharmacology study**

Ho-Sung Lee^1,2^, In-Hee Lee^1^, Kyungrae Kang^2^, Sang-In Park^3^,
Tae-Wook Kwon^2^, and Dae-Yeon Lee^1,2,*^

^1^The Fore, 87 Ogeum-ro, Songpa-gu, Seoul 05542, Republic of Korea.

^2^Forest Hospital, 129 Ogeum-ro, Songpa-gu, Seoul 05549, Republic of Korea.

^3^Forestheal Hospital, 173 Ogeum-ro, Songpa-gu, Seoul 05641, Republic of Korea.

^*^Correspondence should be addressed to Dae-Yeon Lee; foresthrnd@gmail.com

## Supplementary Figures


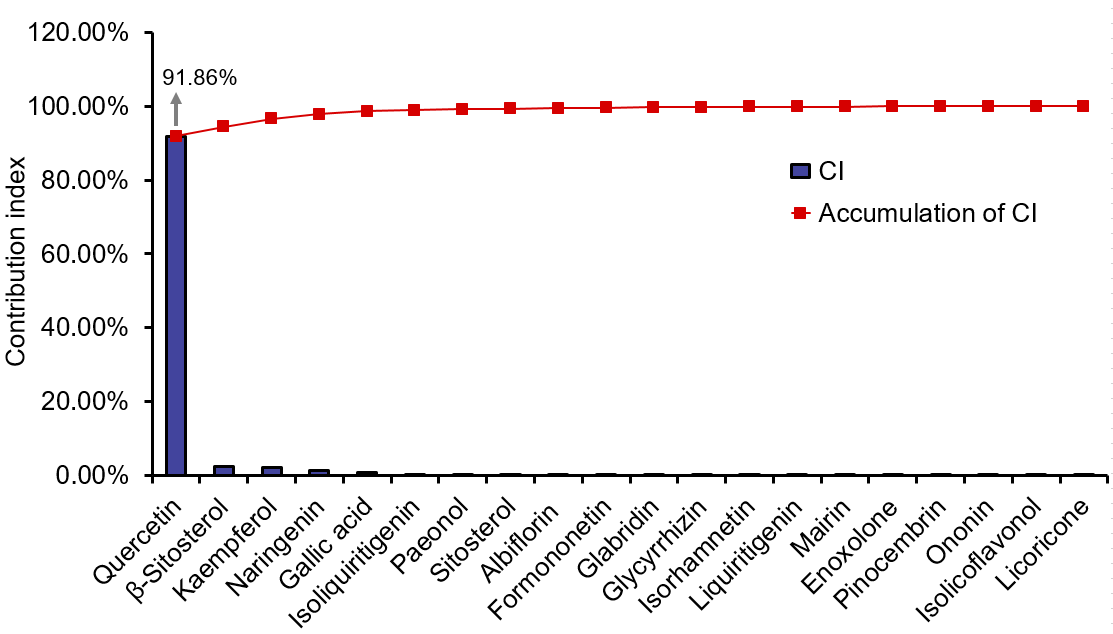


**Supplementary Figure S1. Contribution index analysis for the analgesic activity of active chemical compounds of Jakyak-Gamcho decoction.** A graph depicting the analysis result of contribution index (CI) for the analgesic activity of active chemical compounds of Jakyak-Gamcho decoction. The CI of quercetin was found to be higher than 85%. Note that the CIs of compounds that are not present in the graph is ‘0’.

**Supplementary Figure S2. Functional enrichment analyses for the pain-associated targets of Jakyak-Gamcho decoction.**

**Supplementary Figure S3. Functional interaction analysis of the pain-associated targets of Jakyak-Gamcho decoction.** Black nodes, pain-associated targets; colored edges, mechanisms of the function interactions between the targets.

## Supplementary Tables

**Supplementary Table S1. List of the chemical compounds contained in Jakyak-Gamcho decoction.**

| Herbal medicines | Chemical compounds | OB | Caco-2 | DL |
| --- | --- | --- | --- | --- |
| PlP | (-)-alpha-cedrene | 55.56 | 1.81 | 0.1 |
| PlP | ()-trans-Myrtanol | 49.66 | 1.17 | 0.06 |
| PlP | (+)-Catechin | 54.83 | -0.03 | 0.24 |
| PlP | (1R)-()-Nopinone | 57.86 | 1.23 | 0.05 |
| PlP | (3aR,6S,7aR)-6-hydroxy-6-methyl-3-methylene-3a,4,7,7a-tetrahydrobenzofuran-2,5-dione | 97.79 | -0.01 | 0.08 |
| PlP | (3R,3aR,6S,7aR)-6-hydroxy-3,6-dimethyl-3a,4,7,7a-tetrahydro-3H-benzofuran-2,5-dione | 104.94 | -0.08 | 0.08 |
| PlP | (3S,3aR,5S,6S,7aR)-5,6-dihydroxy-3,6-dimethyl-3,3a,4,5,7,7a-hexahydrobenzofuran-2-one | 96.64 | -0.16 | 0.07 |
| PlP | (3S,5R,8R,9R,10S,14S)-3,17-dihydroxy-4,4,8,10,14-pentamethyl-2,3,5,6,7,9-hexahydro-1H-cyclopenta[a]phenanthrene-15,16-dione  {Palbinone} | 43.56 | 0 | 0.53 |
| PlP | (6R,10R)-6,10,14-trimethylpentadecan-2-one | 23.3 | 1.41 | 0.1 |
| PlP | (6R,10R)-6,10,14-trimethylpentadecan-2-one | 23.3 | 1.41 | 0.1 |
| PlP | (Z)-(1S,5R)-beta-pinen-10-yl-beta-vicianoside | 5.74 | -1.56 | 0.67 |
| PlP | (Z)-(1S,5R)-beta-pinen-10-yl-beta-vicianoside_qt | 50.32 | 1.52 | 0.06 |
| PlP | [(3S,3aR,6S,7aR)-6-hydroxy-6-methyl-2,5-dioxo-3a,4,7,7a-tetrahydro-3H-benzofuran-3-yl]methyl benzoate | 17.84 | -0.17 | 0.3 |
| PlP | 1,2,3,6-tetra-O-galloylglucose | 3.01 | -2.97 | 0.34 |
| PlP | 10-Methylnonadecane | 10.28 | 1.84 | 0.12 |
| PlP | 10-Methylnonadecane | 10.28 | 1.84 | 0.12 |
| PlP | 11alpha,12alpha-epoxy-3beta-23-dihydroxy-30-norolean-20-en-28,12beta-olide | 64.77 | 0.09 | 0.38 |
| PlP | 2 - methyl - 3 - (2 - propenyl) - phenol | 52.06 | 1.64 | 0.03 |
| PlP | 2,2-dimethylcyclohexanol | 82.54 | 1.22 | 0.02 |
| PlP | 24253-30-3 | 74.2 | 1.33 | 0.01 |
| PlP | 24-Methylenecycloartanol | 10.4 | 1.42 | 0.79 |
| PlP | 2-Hexyl-1-decanol | 17.08 | 1.29 | 0.07 |
| PlP | 3,4,5-trihydroxybenzoic acid {Gallic acid} | 31.69 | -0.09 | 0.04 |
| PlP | 3β,23-dihydroxy-oleana-11,13(18)-dien-28-oic acid | 21.53 | 0.1 | 0.75 |
| PlP | 3β-hydroxy-11-oxo-olean-12-en-28-oic acid | 13.49 | 0.21 | 0.74 |
| PlP | 3β-hydroxy-oleana-11,13(18)-dien-28-oic acid? | 17.11 | 0.55 | 0.76 |
| PlP | 4-Chlorobutyric acid | 85.82 | 0.74 | 0.01 |
| PlP | 9-methylenefluorene | 26.87 | 1.95 | 0.09 |
| PlP | acetic acid | 47.87 | 0.42 | 0 |
| PlP | acetic acid | 47.87 | 0.42 | 0 |
| PlP | Acetyl oxide | 45.13 | 0.65 | 0.01 |
| PlP | Albiflorin | 12.09 | -1.54 | 0.77 |
| PlP | Albiflorin R1 | 21.29 | -1.53 | 0.82 |
| PlP | Albiflorin R1_qt | 26.18 | -0.46 | 0.34 |
| PlP | Albiflorin_qt | 66.64 | -0.49 | 0.33 |
| PlP | alexandrin | 20.63 | -0.29 | 0.62 |
| PlP | Astragalin | 14.03 | -1.34 | 0.74 |
| PlP | Benzoylpaeoniflorin | 31.27 | -0.69 | 0.75 |
| PlP | β-Sitosterol | 36.91 | 1.32 | 0.75 |
| PlP | Bicetyl | 8.03 | 1.96 | 0.46 |
| PlP | bicyclo[3.1.1]hept-2-ene-2-methanol, 6,6-dimethyl- | 49.79 | 1.23 | 0.06 |
| PlP | BOX | 31.55 | 0.54 | 0.02 |
| PlP | BU3 | 34.87 | 0.19 | 0.01 |
| PlP | Cedrol | 16.23 | 1.35 | 0.12 |
| PlP | Cedrol | 16.23 | 1.35 | 0.12 |
| PlP | cis-5-Octen-1-ol | 31.84 | 1.16 | 0.01 |
| PlP | DBP | 64.54 | 0.8 | 0.13 |
| PlP | Dibutylphenol | 38.9 | 1.73 | 0.06 |
| PlP | Dipropyl phthalate | 66.3 | 0.78 | 0.1 |
| PlP | Dodecanal | 21.52 | 1.4 | 0.03 |
| PlP | EEE | 45.02 | 1.07 | 0 |
| PlP | Ethylisobutyrate | 83.67 | 1.24 | 0.01 |
| PlP | Ethylisobutyrate | 83.67 | 1.24 | 0.01 |
| PlP | gallotannin | 7.36 | -5.47 | 0.03 |
| PlP | galloylpaeoniflorin | 3.03 | -1.77 | 0.42 |
| PlP | Hederagenol | 22.42 | 0.1 | 0.74 |
| PlP | Henicosane | 8.41 | 1.84 | 0.15 |
| PlP | Heptadekan | 8.64 | 1.84 | 0.07 |
| PlP | Lactiflorin | 49.12 | -1.13 | 0.8 |
| PlP | LFA | 8.46 | 1.83 | 0.13 |
| PlP | Methyl linolelaidate | 41.93 | 1.46 | 0.17 |
| PlP | Methylgallate | 30.91 | 0.26 | 0.05 |
| PlP | myristic acid | 21.18 | 1.07 | 0.07 |
| PlP | Octacosane | 8.15 | 1.91 | 0.37 |
| PlP | octadec-9-ene | 19.5 | 1.87 | 0.09 |
| PlP | oleanolic acid | 29.02 | 0.59 | 0.76 |
| PlP | Oxypaeoniflorin | 21.88 | -1.88 | 0.78 |
| PlP | Paeoniflorigenone | 87.59 | -0.09 | 0.37 |
| PlP | Paeoniflorin | 53.87 | -1.47 | 0.79 |
| PlP | Paeoniflorin_qt | 68.18 | -0.34 | 0.4 |
| PlP | Paeonol | 28.79 | 0.93 | 0.04 |
| PlP | paeonoside | 3.47 | -2.71 | 0.71 |
| PlP | PENTADECYLIC ACID | 20.18 | 1.08 | 0.08 |
| PlP | Pentagalloylglucose | 3.01 | -3.08 | 0.21 |
| PlP | Pisol | 18.5 | 1.23 | 0.03 |
| PlP | Progallin A | 25.61 | 0.33 | 0.06 |
| PlP | propyl (2R)-2-hydroxypropanoate | 25.5 | 0.44 | 0.01 |
| PlP | Pulchinenoside A_qt | 16.91 | 0.12 | 0.77 |
| PlP | PYG | 22.98 | 0.69 | 0.02 |
| PlP | salicylic acid | 32.13 | 0.63 | 0.03 |
| PlP | Satol | 27.27 | 1.34 | 0.11 |
| PlP | Satol | 27.27 | 1.34 | 0.11 |
| PlP | Sitogluside | 20.63 | -0.14 | 0.62 |
| PlP | Sitogluside | 20.63 | -0.14 | 0.62 |
| PlP | stearic acid | 17.83 | 1.15 | 0.14 |
| PlP | sucrose | 7.17 | -2.89 | 0.23 |
| PlP | sucrose | 7.17 | -2.89 | 0.23 |
| PlP | TRD | 17.89 | 1.78 | 0.03 |
| PlP | ZINC02169908 | 23.3 | 1.42 | 0.1 |
| PlP/GuF | Kaempferol | 41.88 | 0.26 | 0.24 |
| PlP/GuF | Mairin | 55.38 | 0.73 | 0.78 |
| PlP/GuF | Sitosterol | 36.91 | 1.32 | 0.75 |
| GuF | (-)-Medicocarpin | 40.99 | -0.6 | 0.95 |
| GuF | ()-Menthol | 59.33 | 1.27 | 0.03 |
| GuF | (1S,2S)-1,2-dimethylcyclopentane | 41.78 | 1.78 | 0.01 |
| GuF | (2R)-1-[2,4-dihydroxy-5-(3-methylbut-2-enyl)phenyl]-2-hydroxy-3-[4-hydroxy-3-(3-methylbut-2-enyl)phenyl]propan-1-one | 1.06 | 0.33 | 0.48 |
| GuF | (2R)-2-[3,4-dihydroxy-5-(3-methylbut-2-enyl)phenyl]-5,7-dihydroxy-8-(3-methylbut-2-enyl)chroman-4-one | 1.21 | 0.51 | 0.63 |
| GuF | (2R)-7-hydroxy-2-(4-hydroxyphenyl)chroman-4-one {ZINC519174} | 71.12 | 0.41 | 0.18 |
| GuF | (2R)-7-hydroxy-2-[4-hydroxy-3-(3-methylbut-2-enyl)phenyl]chroman-4-one | 5.99 | 0.74 | 0.33 |
| GuF | (2S)-2-[4-hydroxy-3-(3-methylbut-2-enyl)phenyl]-8,8-dimethyl-2,3-dihydropyrano[2,3-f]chromen-4-one {Shinflavanone} | 31.79 | 1 | 0.72 |
| GuF | (2S)-6-(2,4-dihydroxyphenyl)-2-(2-hydroxypropan-2-yl)-4-methoxy-2,3-dihydrofuro[3,2-g]chromen-7-one | 60.25 | 0 | 0.63 |
| GuF | (2S)-7-hydroxy-2-(4-hydroxyphenyl)-8-(3-methylbut-2-enyl)chroman-4-one {Isobavachin} | 36.57 | 0.72 | 0.32 |
| GuF | (3S)-2,3-dimethylpentane | 35.57 | 1.78 | 0.01 |
| GuF | (4S)-2,4-dimethylhexane | 37.13 | 1.77 | 0.01 |
| GuF | (E)-1-(2,4-dihydroxyphenyl)-3-(2,2-dimethylchromen-6-yl)prop-2-en-1-one {Kanzonol B} | 39.62 | 0.66 | 0.35 |
| GuF | (E)-1-(2,4-dihydroxyphenyl)-3-[4-hydroxy-3-(3-methylbut-2-enyl)phenyl]prop-2-en-1-one | 1.04 | 0.55 | 0.27 |
| GuF | (E)-1-[2,4-dihydroxy-3-(3-methylbut-2-enyl)phenyl]-3-(2,4-dihydroxyphenyl)prop-2-en-1-one | 1.36 | 0.5 | 0.3 |
| GuF | (E)-1-[2,4-dihydroxy-3-(3-methylbut-2-enyl)phenyl]-3-[4-hydroxy-3-(3-methylbut-2-enyl)phenyl]prop-2-en-1-one | 1.02 | 0.81 | 0.45 |
| GuF | (E)-1-butoxyhex-2-ene | 41.72 | 1.5 | 0.02 |
| GuF | (E)-3-[3,4-dihydroxy-5-(3-methylbut-2-enyl)phenyl]-1-(2,4-dihydroxyphenyl)prop-2-en-1-one | 46.27 | 0.41 | 0.31 |
| GuF | (E)-dodec-2-ene | 17.74 | 1.83 | 0.02 |
| GuF | (L)-alpha-Terpineol | 48.8 | 1.39 | 0.03 |
| GuF | (Z)-1-(2,4-dihydroxyphenyl)-3-phenylprop-2-en-1-one | 73.18 | 0.57 | 0.12 |
| GuF | 1-(5-hydroxy-2,2-dimethylchromen-6-yl)-3-(4-hydroxyphenyl)prop-2-en-1-one | 5.2 | 0.86 | 0.34 |
| GuF | 1,3-dihydroxy-8,9-dimethoxy-6-benzofurano[3,2-c]chromenone  {1,3-Dihydroxy-8,9-dimethoxy-[1]benzofuro[3,2-c]chromen-6-one} | 62.9 | 0.4 | 0.53 |
| GuF | 1,3-dihydroxy-9-methoxy-6-benzofurano[3,2-c]chromenone {Hedysarimcoumestan B} | 48.14 | 0.48 | 0.43 |
| GuF | 11-deoxyglycyrrhetic acid | 16.21 | 0.51 | 0.76 |
| GuF | 12-methyltetradecanoate | 17.36 | 1.35 | 0.09 |
| GuF | 18beta-glycyrrhetinic acid {Enoxolone} | 22.05 | 0.1 | 0.74 |
| GuF | 18α-hydroxyglycyrrhetic acid | 41.16 | -0.29 | 0.71 |
| GuF | 1-Methoxyficifolinol | 14.61 | 1.09 | 0.86 |
| GuF | 1-Methoxyphaseollidin | 69.98 | 1.01 | 0.64 |
| GuF | 2-(3,4-dihydroxyphenyl)-5,7-dihydroxy-6-(3-methylbut-2-enyl)chromone {Gancaonin O} | 44.15 | 0.48 | 0.41 |
| GuF | 2,2-DIMETHYLPENTANE | 55.33 | 1.79 | 0.01 |
| GuF | 2,3-dimethylhexane | 46.24 | 1.78 | 0.01 |
| GuF | 2,6,10-trimethyl-dodecane | 37.8 | 0.08 | 0.03 |
| GuF | 2',7-Dihydroxy-4'-methoxyisoflavan-7-O-β-d-glucopyranoside | 10.46 | -1.02 | 0.73 |
| GuF | 2-[(3R)-8,8-dimethyl-3,4-dihydro-2H-pyrano[6,5-f]chromen-3-yl]-5-methoxyphenol  {4'-Methoxyglabridin} | 36.21 | 1.12 | 0.52 |
| GuF | 21987_FLUKA | 40.92 | 1.84 | 0.04 |
| GuF | 22β-acetylglabric acid | 17.76 | -0.21 | 0.64 |
| GuF | 24-Hydroxy-11-deoxyglycyrrhetic acid | 17.57 | 0.26 | 0.76 |
| GuF | 24-Hydroxyglycyrrhetic acid | 24.17 | -0.1 | 0.72 |
| GuF | 2-Caren-10-al | 44.74 | 1.37 | 0.05 |
| GuF | 2-Ethyl-p-xylene | 20.6 | 1.89 | 0.02 |
| GuF | 2-heptanone | 46.56 | 1.31 | 0.01 |
| GuF | 2-methyl-5-propyl -nonane | 15.28 | 1.81 | 0.03 |
| GuF | 2-methyl-6-ethyl decane | 5.5 | 1.81 | 0.03 |
| GuF | 2-Tetradecanone | 17.71 | 1.46 | 0.05 |
| GuF | 3-(2,4-dihydroxyphenyl)-8-(1,1-dimethylprop-2-enyl)-7-hydroxy-5-methoxy-coumarin  {Licoarylcoumarin} | 59.62 | 0.4 | 0.43 |
| GuF | 3-(2-hydroxy-4-methoxyphenyl)-2H-chromen-7-ol | 4.66 | 0.89 | 0.21 |
| GuF | 3-(3,4-dihydroxyphenyl)-5,7-dihydroxy-8-(3-methylbut-2-enyl)chromone {Gancaonin L} | 66.37 | 0.52 | 0.41 |
| GuF | 3'(γ,γ-dimethylallyl)-kievitone | 1.21 | 0.51 | 0.63 |
| GuF | 3,22-Dihydroxy-11-oxo-delta(12)-oleanene-27-alpha-methoxycarbonyl-29-oic acid  {Glyuranolide} | 34.32 | -0.06 | 0.55 |
| GuF | 3,3-Dimethylpentane | 41.97 | 1.75 | 0.01 |
| GuF | 3,4,3',4'-Tetrahydroxy-2-methoxychalcone | 1.33 | 0.64 | 0.2 |
| GuF | 3-[4,6-dihydroxy-2-methoxy-3-(3-methylbut-2-enyl)phenyl]-7-hydroxy-chromone | 2.47 | 0.43 | 0.44 |
| GuF | 3-Ethylpentane | 35.74 | 1.79 | 0.01 |
| GuF | 3'-Hydroxy-4'-O-methylglabridin | 43.71 | 1 | 0.57 |
| GuF | 3-Hydroxyglabrol | 4.73 | 0.48 | 0.58 |
| GuF | 3'-Methoxyglabridin | 46.16 | 0.94 | 0.57 |
| GuF | 3-methylheptane | 36.61 | 1.79 | 0.01 |
| GuF | 3-methylhexane | 38.19 | 1.78 | 0.01 |
| GuF | 3-Methylpentane | 35.77 | 1.76 | 0 |
| GuF | 3β-formylglabrolide | 16.36 | 0.26 | 0.55 |
| GuF | 4,2',4',alpha-Tetrahydroxydihydrochalcone | 2.45 | 0.1 | 0.16 |
| GuF | 4H-1-Benzopyran-4-one, 2-(4-(beta-D-glucopyranosyloxy)phenyl)-2,3-dihydro-5,7-dihydroxy-, (2S)- | 14.03 | -1.13 | 0.78 |
| GuF | 5,6,7,8-Tetrahydro-2,4-dimethylquinoline | 49.77 | 1.64 | 0.05 |
| GuF | 5,6,7,8-Tetrahydro-4-methylquinoline | 59.18 | 1.63 | 0.04 |
| GuF | 5,7-dihydroxy-3-(2-hydroxy-4-methoxy-phenyl)-6-(3-methylbut-2-enyl)chromone | 2.47 | 0.58 | 0.45 |
| GuF | 5,7-dihydroxy-3-(4-methoxyphenyl)-8-(3-methylbut-2-enyl)chromone {Gancaonin M} | 30.49 | 0.9 | 0.41 |
| GuF | 6″-O-acetylliquiritin | 6.26 | -0.48 | 0.82 |
| GuF | 6-Prenylated eriodictyol | 39.22 | 0.4 | 0.41 |
| GuF | 7,2',4'-trihydroxy-5-methoxy-3-arylcoumarin {ZINC105741014} | 83.71 | 0.24 | 0.27 |
| GuF | 7,4'-Dihydroxyflavone | 19.18 | 0.56 | 0.18 |
| GuF | 7-Acetoxy-2-methylisoflavone | 38.92 | 0.74 | 0.26 |
| GuF | 7-hydroxy-2-[4-hydroxy-3-(3-methylbut-2-enyl)phenyl]-6-(3-methylbut-2-enyl)chromone | 4.44 | 0.88 | 0.56 |
| GuF | 7-hydroxy-2-methyl-3-phenyl-chromone | 25.8 | 1 | 0.18 |
| GuF | 7-Methoxy-2-methyl isoflavone {ZINC520945} | 42.56 | 1.16 | 0.2 |
| GuF | 8-(6-hydroxy-2-benzofuranyl)-2,2-dimethyl-5-chromenol {Kanzonol U} | 58.44 | 1 | 0.38 |
| GuF | 8-Prenylated eriodictyol | 53.79 | 0.43 | 0.4 |
| GuF | 8-Prenylwighteone | 23.22 | 0.93 | 0.54 |
| GuF | anethole | 32.49 | 1.75 | 0.03 |
| GuF | apioglycyrrhizin | 17.8 | -1.91 | 0.14 |
| GuF | apioglycyrrhizin_qt | 23.73 | 0.1 | 0.74 |
| GuF | Araboglycyrrhizin | 17.73 | -2.46 | 0.14 |
| GuF | Araboglycyrrhizin_qt | 17.71 | 0.11 | 0.74 |
| GuF | Arachic acid | 16.66 | 1.18 | 0.19 |
| GuF | Artonin E | 11.38 | 0.34 | 0.8 |
| GuF | Astragalin | 14.03 | -1.34 | 0.74 |
| GuF | beta-Glycyrrhetinic acid | 17.41 | 0.19 | 0.74 |
| GuF | beta-Terpinene | 42.29 | 1.85 | 0.02 |
| GuF | BuOH | 22.02 | 0.94 | 0 |
| GuF | butylated hydroxytoluene | 40.02 | 1.75 | 0.07 |
| GuF | Calycosin | 47.75 | 0.52 | 0.24 |
| GuF | Castanin | 23.54 | 0.77 | 0.27 |
| GuF | Corylifolinin | 1.04 | 0.81 | 0.27 |
| GuF | Cyclobutanol, 1-ethyl- | 93.23 | 1.13 | 0.02 |
| GuF | Daidzein dimethyl ether | 24.29 | 0.98 | 0.24 |
| GuF | DBP | 64.54 | 0.8 | 0.13 |
| GuF | dehydroglyasperin C | 53.82 | 0.68 | 0.37 |
| GuF | DFV {Liquiritigenin} | 32.76 | 0.51 | 0.18 |
| GuF | DIBP | 49.63 | 0.85 | 0.13 |
| GuF | Docosyl caffeate | 3.14 | 1.01 | 0.59 |
| GuF | EB | 49.38 | 1.83 | 0.01 |
| GuF | echinatin | 66.58 | 0.38 | 0.17 |
| GuF | Euchrenone | 30.29 | 1.09 | 0.57 |
| GuF | Eurycarpin A | 43.28 | 0.43 | 0.37 |
| GuF | Formononetin | 69.67 | 0.78 | 0.21 |
| GuF | Gadelaidic acid | 30.7 | 1.2 | 0.2 |
| GuF | Gancaonin A | 51.08 | 0.8 | 0.4 |
| GuF | Gancaonin B | 48.79 | 0.58 | 0.45 |
| GuF | Gancaonin C | 2.87 | 0.14 | 0.42 |
| GuF | Gancaonin D | 2.72 | -0.11 | 0.51 |
| GuF | Gancaonin G | 60.44 | 0.78 | 0.39 |
| GuF | Gancaonin H | 50.1 | 0.6 | 0.78 |
| GuF | Gancaonin I | 21.9 | 0.93 | 0.39 |
| GuF | Gancaonin P | 1.41 | 0.27 | 0.45 |
| GuF | Gancaonin Q | 8.98 | 0.86 | 0.6 |
| GuF | Gancaonin R | 1.26 | 1.03 | 0.37 |
| GuF | Gancaonin S | 1.26 | 0.98 | 0.38 |
| GuF | gancaonin T | 1.04 | 0.5 | 0.53 |
| GuF | Gancaonin U | 14.53 | 1.08 | 0.53 |
| GuF | Gancaonin V | 1.24 | 0.73 | 0.34 |
| GuF | Glabranin | 52.9 | 0.97 | 0.31 |
| GuF | Glabrene | 46.27 | 0.99 | 0.44 |
| GuF | Glabridin | 53.25 | 0.97 | 0.47 |
| GuF | glabrol | 4.25 | 0.84 | 0.54 |
| GuF | glabrolide | 17.46 | 0.29 | 0.61 |
| GuF | Glabrone | 52.51 | 0.59 | 0.5 |
| GuF | Glepidotin A | 44.72 | 0.79 | 0.35 |
| GuF | Glepidotin B | 64.46 | 0.46 | 0.34 |
| GuF | glucuronic acid | 46.18 | -2.05 | 0.06 |
| GuF | Glyasperin A | 2.46 | 0.6 | 0.63 |
| GuF | glyasperin B | 65.22 | 0.47 | 0.44 |
| GuF | Glyasperin C | 45.56 | 0.71 | 0.4 |
| GuF | glyasperin E | 4.12 | 0.69 | 0.75 |
| GuF | Glyasperin F | 75.84 | 0.43 | 0.54 |
| GuF | Glyasperin M | 72.67 | 0.49 | 0.59 |
| GuF | glyasperins D | 29.91 | 0.89 | 0.43 |
| GuF | Glyasperins K | 10.15 | 0.74 | 0.44 |
| GuF | glyasperins Z | 4.17 | 1.09 | 0.36 |
| GuF | Glycycoumarin | 23.56 | 0.52 | 0.44 |
| GuF | Glycyram | 19.62 | -2.66 | 0.11 |
| GuF | Glycyrin | 52.61 | 0.59 | 0.47 |
| GuF | Glycyrol | 90.78 | 0.71 | 0.67 |
| GuF | glycyroside | 37.25 | -1.58 | 0.79 |
| GuF | glycyrrhetol | 14.66 | 0.36 | 0.75 |
| GuF | Glycyrrhiza flavonol A | 41.28 | -0.09 | 0.6 |
| GuF | Glycyrrhizin | 9.06 | -2.23 | 0.11 |
| GuF | glyinflanin A | 1.06 | 0.69 | 0.48 |
| GuF | Glypallichalcone | 61.6 | 0.76 | 0.19 |
| GuF | Glyzaglabrin | 61.07 | 0.34 | 0.35 |
| GuF | Heptan | 41.8 | 1.77 | 0 |
| GuF | HEX | 52.5 | 1.78 | 0 |
| GuF | Hirsutrin | 1.86 | -1.66 | 0.77 |
| GuF | Hispaglabridin A | 14.6 | 1.12 | 0.73 |
| GuF | Hispaglabridin B | 22.94 | 1.18 | 0.88 |
| GuF | HMO {Isoformononetin} | 38.37 | 0.79 | 0.21 |
| GuF | ICO | 33.86 | 0.82 | 0.05 |
| GuF | icos-5-enoic acid {5E-Eicosenoic acid} | 30.7 | 1.22 | 0.2 |
| GuF | Inermine | 75.18 | 0.89 | 0.54 |
| GuF | Inflacoumarin A | 39.71 | 0.73 | 0.33 |
| GuF | isoglabrolide | 14.77 | 0.32 | 0.62 |
| GuF | isoglycycoumarin | 22.09 | 0.55 | 0.6 |
| GuF | Isoglycyrol | 44.7 | 0.91 | 0.84 |
| GuF | isograbrol | 11.04 | 0.87 | 0.5 |
| GuF | ISOHEPTANE | 59.94 | 1.81 | 0.01 |
| GuF | Isohexane | 56.13 | 1.77 | 0 |
| GuF | Isolicoflavonol | 45.17 | 0.54 | 0.42 |
| GuF | isoliquiritigenin | 85.32 | 0.44 | 0.15 |
| GuF | Isoliquiritin | 8.61 | -1.36 | 0.6 |
| GuF | Isoononin | 8.29 | -1 | 0.79 |
| GuF | Isorhamnetin | 49.6 | 0.31 | 0.31 |
| GuF | Isoschaftoside | 17.38 | -2.62 | 0.83 |
| GuF | Isotrifoliol | 31.94 | 0.53 | 0.42 |
| GuF | Isoviolanthin | 18.79 | -2.43 | 0.81 |
| GuF | Izoforon | 44.98 | 1.28 | 0.03 |
| GuF | Jaranol | 50.83 | 0.61 | 0.29 |
| GuF | Kanzonol E | 5.77 | 0.98 | 0.71 |
| GuF | Kanzonol F | 32.47 | 1.18 | 0.89 |
| GuF | Kanzonol H | 16.92 | 0.96 | 0.8 |
| GuF | Kanzonol Z | 21.77 | 0.5 | 0.76 |
| GuF | kanzonols K | 0.97 | 0.76 | 0.66 |
| GuF | kanzonols L | 0.98 | 0.7 | 0.78 |
| GuF | kanzonols T | 17.87 | 0.05 | 0.67 |
| GuF | kanzonols W {Kanzonol W} | 50.48 | 0.63 | 0.52 |
| GuF | kanzonols X | 7.56 | 1.1 | 0.56 |
| GuF | Karenzu DK2 | 62.26 | 0.94 | 0.1 |
| GuF | Licoagrocarpin | 58.81 | 1.23 | 0.58 |
| GuF | Licoagroisoflavone | 57.28 | 0.71 | 0.49 |
| GuF | licoagropin | 27.14 | 1.63 | 0.51 |
| GuF | Licochalcone A | 40.79 | 0.82 | 0.29 |
| GuF | Licochalcone B | 76.76 | 0.47 | 0.19 |
| GuF | licochalcone C | 4.44 | 0.63 | 0.29 |
| GuF | Licochalcone G | 49.25 | 0.64 | 0.32 |
| GuF | licochalconeD | 1.01 | 0.47 | 0.34 |
| GuF | Licocoumarone | 33.21 | 0.84 | 0.36 |
| GuF | Licoflavone | 18.75 | 0.82 | 0.33 |
| GuF | Licoflavonol | 8.75 | 0.49 | 0.4 |
| GuF | Licoisoflavanone | 52.47 | 0.39 | 0.54 |
| GuF | Licoisoflavone | 41.61 | 0.37 | 0.42 |
| GuF | Licoisoflavone B | 38.93 | 0.46 | 0.55 |
| GuF | Liconeolignan | 4.41 | 1 | 0.4 |
| GuF | licopyranocoumarin | 80.36 | 0.13 | 0.65 |
| GuF | Licorice glycoside A | 5.95 | -2.37 | 0.35 |
| GuF | licorice glycoside E | 32.89 | -2.06 | 0.27 |
| GuF | licorice-saponin B2 | 58.55 | -2.4 | 0.11 |
| GuF | licorice-saponin C2 | 59.66 | -2.28 | 0.11 |
| GuF | licorice-saponin C2_qt | 17.33 | 0.48 | 0.76 |
| GuF | licorice-saponin F3 | 17.68 | -2.82 | 0.03 |
| GuF | licorice-saponin F3_qt | 27.53 | 0.74 | 0.64 |
| GuF | licorice-saponin G2 | 6.39 | -2.01 | 0.11 |
| GuF | licorice-saponin G2_qt | 22.78 | -0.27 | 0.72 |
| GuF | licorice-saponin H2 | 44.37 | -2.08 | 0.11 |
| GuF | licorice-saponin H2_qt | 22.91 | 0.01 | 0.74 |
| GuF | licorice-saponin J2 | 6.25 | -2.25 | 0.11 |
| GuF | licorice-saponin J2_qt | 28.3 | 0.04 | 0.74 |
| GuF | licorice-saponin K2 | 7.82 | -2.64 | 0.11 |
| GuF | licorice-saponin K2_qt | 27.79 | 0.05 | 0.75 |
| GuF | Licoricidin | 0.99 | 0.96 | 0.62 |
| GuF | Licoricone | 63.58 | 0.53 | 0.47 |
| GuF | Licoriisoflavan A | 3.68 | 1.1 | 0.66 |
| GuF | Licuraside | 5.25 | -1.92 | 0.77 |
| GuF | Liquiritin | 65.69 | -1.06 | 0.74 |
| GuF | Liquiritin apioside | 29.23 | -1.88 | 0.82 |
| GuF | liquoric acid | 25.44 | -0.01 | 0.55 |
| GuF | Lupiwighteone | 51.64 | 0.68 | 0.37 |
| GuF | Medicarpin | 49.22 | 1 | 0.34 |
| GuF | Methylcyclopentane | 55.78 | 1.79 | 0.01 |
| GuF | Methylheptane | 28.65 | 1.79 | 0.01 |
| GuF | Mipax | 57.4 | 0.64 | 0.06 |
| GuF | Morusin | 11.52 | 0.51 | 0.76 |
| GuF | m-xylene | 47.43 | 1.83 | 0.01 |
| GuF | Narcissoside | 5.09 | -2.14 | 0.65 |
| GuF | Naringenin | 59.29 | 0.28 | 0.21 |
| GuF | naringin | 6.92 | -1.99 | 0.78 |
| GuF | neoisoliquiritin | 21.18 | -1.41 | 0.58 |
| GuF | neoliquiritin | 13.01 | -1.08 | 0.71 |
| GuF | Neouralenol | 12.76 | 0.24 | 0.46 |
| GuF | nicotiflorin | 3.64 | -1.77 | 0.73 |
| GuF | Nortangeretin | 17.9 | 0.24 | 0.27 |
| GuF | OCT | 29.72 | 1.78 | 0.01 |
| GuF | Octadiene | 34.53 | 1.81 | 0.01 |
| GuF | Odoratin | 49.95 | 0.42 | 0.3 |
| GuF | oleanolic acid | 29.02 | 0.59 | 0.76 |
| GuF | Ononin | 11.52 | -0.74 | 0.78 |
| GuF | o-xylene | 45.55 | 1.85 | 0.01 |
| GuF | Pentadecanol | 13.73 | 1.3 | 0.06 |
| GuF | PENTYLFURAN | 54.59 | 1.72 | 0.02 |
| GuF | Phaseol | 78.77 | 0.76 | 0.58 |
| GuF | Phaseolinisoflavan | 32.01 | 1.01 | 0.45 |
| GuF | Pinocembrin | 64.72 | 0.61 | 0.18 |
| GuF | protocatechuic acid | 25.37 | 0.1 | 0.04 |
| GuF | Prunetin | 5.41 | 0.65 | 0.24 |
| GuF | p-xylene | 48.74 | 1.83 | 0.01 |
| GuF | Quercetin | 46.43 | 0.05 | 0.28 |
| GuF | Quercetin der. {Quercetin 3,3'-dimethyl ether} | 46.45 | 0.39 | 0.33 |
| GuF | rutin | 3.2 | -1.93 | 0.68 |
| GuF | schaftoside | 7.88 | -2.46 | 0.75 |
| GuF | Scopoletol | 27.77 | 0.71 | 0.08 |
| GuF | Semilicoisoflavone B | 48.78 | 0.45 | 0.55 |
| GuF | Sextone B | 56.2 | 1.77 | 0.01 |
| GuF | Shinpterocarpin | 80.3 | 1.1 | 0.73 |
| GuF | Sigmoidin B | 34.88 | 0.42 | 0.41 |
| GuF | Uralene | 11.7 | 0.63 | 0.49 |
| GuF | uralenneoside | 24.96 | -1.01 | 0.17 |
| GuF | Uralenol | 8.55 | 0.35 | 0.46 |
| GuF | Uralenol-3-methylether | 1.41 | 0.39 | 0.49 |
| GuF | uralsaponin B | 7.92 | -2.6 | 0.11 |
| GuF | ursolic acid | 16.77 | 0.67 | 0.75 |
| GuF | Vestitol | 74.66 | 0.86 | 0.21 |
| GuF | Vicenin-2 | 3.42 | -3.15 | 0.78 |
| GuF | violanthin | 4.17 | -2.01 | 0.81 |
| GuF | vitexin | 3.05 | -1.52 | 0.71 |
| GuF | WLN: 4OVR | 48.41 | 1.31 | 0.04 |
| GuF | WLN: VH6 | 19.59 | 1.29 | 0.01 |
| GuF | Xambioona | 54.85 | 1.09 | 0.87 |
| GuF | Yinyanghuo D | 13.99 | 0.61 | 0.38 |
| GuF | α-cubebol | 64.81 | 1.32 | 0.09 |

PlP, *Paeonia lactiflora* Pallas; GuF, *Glycyrrhiza uralensis* Fischer; OB, oral bioavailability; Caco-2, Caco-2 cell permeability; DL, drug-likeness score. Synonyms of certain chemical compounds are given in braces {}.

**Supplementary Table S2. List of the active chemical compounds in Jakyak-Gamcho decoction.**

| Herbal medicines | Chemical compounds | OB | Caco-2 | DL |
| --- | --- | --- | --- | --- |
| PlP | (+)-Catechin | 54.83 | -0.03 | 0.24 |
| PlP | (3S,5R,8R,9R,10S,14S)-3,17-dihydroxy-4,4,8,10,14-pentamethyl-2,3,5,6,7,9-hexahydro-1H-cyclopenta[a]phenanthrene-15,16-dione  {Palbinone} | 43.56 | 0 | 0.53 |
| PlP | 11alpha,12alpha-epoxy-3beta-23-dihydroxy-30-norolean-20-en-28,12beta-olide | 64.77 | 0.09 | 0.38 |
| PlP | 3,4,5-trihydroxybenzoic acid {Gallic acid} | 31.69 | -0.09 | 0.04 |
| PlP | Albiflorin | 12.09 | -1.54 | 0.77 |
| PlP | β-Sitosterol | 36.91 | 1.32 | 0.75 |
| PlP | Oxypaeoniflorin | 21.88 | -1.88 | 0.78 |
| PlP | Paeoniflorigenone | 87.59 | -0.09 | 0.37 |
| PlP | Paeoniflorin | 53.87 | -1.47 | 0.79 |
| PlP | Paeonol | 28.79 | 0.93 | 0.04 |
| PlP/GuF | Kaempferol | 41.88 | 0.26 | 0.24 |
| PlP/GuF | Mairin | 55.38 | 0.73 | 0.78 |
| PlP/GuF | Sitosterol | 36.91 | 1.32 | 0.75 |
| GuF | (2R)-7-hydroxy-2-(4-hydroxyphenyl)chroman-4-one {ZINC519174} | 71.12 | 0.41 | 0.18 |
| GuF | (2S)-2-[4-hydroxy-3-(3-methylbut-2-enyl)phenyl]-8,8-dimethyl-2,3-dihydropyrano[2,3-f]chromen-4-one  {Shinflavanone} | 31.79 | 1 | 0.72 |
| GuF | (2S)-6-(2,4-dihydroxyphenyl)-2-(2-hydroxypropan-2-yl)-4-methoxy-2,3-dihydrofuro[3,2-g]chromen-7-one | 60.25 | 0 | 0.63 |
| GuF | (2S)-7-hydroxy-2-(4-hydroxyphenyl)-8-(3-methylbut-2-enyl)chroman-4-one {Isobavachin} | 36.57 | 0.72 | 0.32 |
| GuF | (E)-1-(2,4-dihydroxyphenyl)-3-(2,2-dimethylchromen-6-yl)prop-2-en-1-one {Kanzonol B} | 39.62 | 0.66 | 0.35 |
| GuF | (E)-3-[3,4-dihydroxy-5-(3-methylbut-2-enyl)phenyl]-1-(2,4-dihydroxyphenyl)prop-2-en-1-one | 46.27 | 0.41 | 0.31 |
| GuF | 1,3-dihydroxy-8,9-dimethoxy-6-benzofurano[3,2-c]chromenone  {1,3-Dihydroxy-8,9-dimethoxy-[1]benzofuro[3,2-c]chromen-6-one} | 62.9 | 0.4 | 0.53 |
| GuF | 1,3-dihydroxy-9-methoxy-6-benzofurano[3,2-c]chromenone {Hedysarimcoumestan B} | 48.14 | 0.48 | 0.43 |
| GuF | 18beta-glycyrrhetinic acid {Enoxolone} | 22.05 | 0.1 | 0.74 |
| GuF | 18α-hydroxyglycyrrhetic acid | 41.16 | -0.29 | 0.71 |
| GuF | 1-Methoxyphaseollidin | 69.98 | 1.01 | 0.64 |
| GuF | 2-(3,4-dihydroxyphenyl)-5,7-dihydroxy-6-(3-methylbut-2-enyl)chromone {Gancaonin O} | 44.15 | 0.48 | 0.41 |
| GuF | 2-[(3R)-8,8-dimethyl-3,4-dihydro-2H-pyrano[6,5-f]chromen-3-yl]-5-methoxyphenol  {4'-Methoxyglabridin} | 36.21 | 1.12 | 0.52 |
| GuF | 3-(2,4-dihydroxyphenyl)-8-(1,1-dimethylprop-2-enyl)-7-hydroxy-5-methoxy-coumarin  {Licoarylcoumarin} | 59.62 | 0.4 | 0.43 |
| GuF | 3-(3,4-dihydroxyphenyl)-5,7-dihydroxy-8-(3-methylbut-2-enyl)chromone {Gancaonin L} | 66.37 | 0.52 | 0.41 |
| GuF | 3,22-Dihydroxy-11-oxo-delta(12)-oleanene-27-alpha-methoxycarbonyl-29-oic acid  {Glyuranolide} | 34.32 | -0.06 | 0.55 |
| GuF | 3'-Hydroxy-4'-O-methylglabridin | 43.71 | 1 | 0.57 |
| GuF | 3'-Methoxyglabridin | 46.16 | 0.94 | 0.57 |
| GuF | 5,7-dihydroxy-3-(4-methoxyphenyl)-8-(3-methylbut-2-enyl)chromone {Gancaonin M} | 30.49 | 0.9 | 0.41 |
| GuF | 6-Prenylated eriodictyol | 39.22 | 0.4 | 0.41 |
| GuF | 7,2',4'-trihydroxy－5-methoxy-3－arylcoumarin {ZINC105741014} | 83.71 | 0.24 | 0.27 |
| GuF | 7-Acetoxy-2-methylisoflavone | 38.92 | 0.74 | 0.26 |
| GuF | 7-Methoxy-2-methyl isoflavone {ZINC520945} | 42.56 | 1.16 | 0.2 |
| GuF | 8-(6-hydroxy-2-benzofuranyl)-2,2-dimethyl-5-chromenol {Kanzonol U} | 58.44 | 1 | 0.38 |
| GuF | 8-Prenylated eriodictyol | 53.79 | 0.43 | 0.4 |
| GuF | Calycosin | 47.75 | 0.52 | 0.24 |
| GuF | dehydroglyasperin C | 53.82 | 0.68 | 0.37 |
| GuF | DFV {Liquiritigenin} | 32.76 | 0.51 | 0.18 |
| GuF | Euchrenone | 30.29 | 1.09 | 0.57 |
| GuF | Eurycarpin A | 43.28 | 0.43 | 0.37 |
| GuF | Formononetin | 69.67 | 0.78 | 0.21 |
| GuF | Gadelaidic acid | 30.7 | 1.2 | 0.2 |
| GuF | Gancaonin A | 51.08 | 0.8 | 0.4 |
| GuF | Gancaonin B | 48.79 | 0.58 | 0.45 |
| GuF | Gancaonin G | 60.44 | 0.78 | 0.39 |
| GuF | Gancaonin H | 50.1 | 0.6 | 0.78 |
| GuF | Glabranin | 52.9 | 0.97 | 0.31 |
| GuF | Glabrene | 46.27 | 0.99 | 0.44 |
| GuF | Glabridin | 53.25 | 0.97 | 0.47 |
| GuF | Glabrone | 52.51 | 0.59 | 0.5 |
| GuF | Glepidotin A | 44.72 | 0.79 | 0.35 |
| GuF | Glepidotin B | 64.46 | 0.46 | 0.34 |
| GuF | glyasperin B | 65.22 | 0.47 | 0.44 |
| GuF | Glyasperin C | 45.56 | 0.71 | 0.4 |
| GuF | Glyasperin F | 75.84 | 0.43 | 0.54 |
| GuF | Glyasperin M | 72.67 | 0.49 | 0.59 |
| GuF | Glycyrin | 52.61 | 0.59 | 0.47 |
| GuF | Glycyrol | 90.78 | 0.71 | 0.67 |
| GuF | Glycyrrhiza flavonol A | 41.28 | -0.09 | 0.6 |
| GuF | Glycyrrhizin | 9.06 | -2.23 | 0.11 |
| GuF | Glypallichalcone | 61.6 | 0.76 | 0.19 |
| GuF | Glyzaglabrin | 61.07 | 0.34 | 0.35 |
| GuF | HMO {Isoformononetin} | 38.37 | 0.79 | 0.21 |
| GuF | icos-5-enoic acid {5E-Eicosenoic acid} | 30.7 | 1.22 | 0.2 |
| GuF | Inermine | 75.18 | 0.89 | 0.54 |
| GuF | Inflacoumarin A | 39.71 | 0.73 | 0.33 |
| GuF | Isoglycyrol | 44.7 | 0.91 | 0.84 |
| GuF | Isolicoflavonol | 45.17 | 0.54 | 0.42 |
| GuF | isoliquiritigenin | 85.32 | 0.44 | 0.15 |
| GuF | Isoliquiritin | 8.61 | -1.36 | 0.6 |
| GuF | Isorhamnetin | 49.6 | 0.31 | 0.31 |
| GuF | Isotrifoliol | 31.94 | 0.53 | 0.42 |
| GuF | Jaranol | 50.83 | 0.61 | 0.29 |
| GuF | Kanzonol F | 32.47 | 1.18 | 0.89 |
| GuF | kanzonols W {Kanzonol W} | 50.48 | 0.63 | 0.52 |
| GuF | Licoagrocarpin | 58.81 | 1.23 | 0.58 |
| GuF | Licoagroisoflavone | 57.28 | 0.71 | 0.49 |
| GuF | Licochalcone A | 40.79 | 0.82 | 0.29 |
| GuF | Licochalcone B | 76.76 | 0.47 | 0.19 |
| GuF | Licochalcone G | 49.25 | 0.64 | 0.32 |
| GuF | Licocoumarone | 33.21 | 0.84 | 0.36 |
| GuF | Licoisoflavanone | 52.47 | 0.39 | 0.54 |
| GuF | Licoisoflavone | 41.61 | 0.37 | 0.42 |
| GuF | Licoisoflavone B | 38.93 | 0.46 | 0.55 |
| GuF | licopyranocoumarin | 80.36 | 0.13 | 0.65 |
| GuF | Licoricone | 63.58 | 0.53 | 0.47 |
| GuF | Liquiritin | 65.69 | -1.06 | 0.74 |
| GuF | Lupiwighteone | 51.64 | 0.68 | 0.37 |
| GuF | Medicarpin | 49.22 | 1 | 0.34 |
| GuF | Naringenin | 59.29 | 0.28 | 0.21 |
| GuF | Odoratin | 49.95 | 0.42 | 0.3 |
| GuF | Ononin | 11.52 | -0.74 | 0.78 |
| GuF | Phaseol | 78.77 | 0.76 | 0.58 |
| GuF | Phaseolinisoflavan | 32.01 | 1.01 | 0.45 |
| GuF | Pinocembrin | 64.72 | 0.61 | 0.18 |
| GuF | Quercetin | 46.43 | 0.05 | 0.28 |
| GuF | Quercetin der. {Quercetin 3,3'-dimethyl ether} | 46.45 | 0.39 | 0.33 |
| GuF | Semilicoisoflavone B | 48.78 | 0.45 | 0.55 |
| GuF | Shinpterocarpin | 80.3 | 1.1 | 0.73 |
| GuF | Sigmoidin B | 34.88 | 0.42 | 0.41 |
| GuF | Vestitol | 74.66 | 0.86 | 0.21 |
| GuF | Xambioona | 54.85 | 1.09 | 0.87 |

PlP, *Paeonia lactiflora* Pallas; GuF, *Glycyrrhiza uralensis* Fischer; OB, oral bioavailability; Caco-2, Caco-2 cell permeability; DL, drug-likeness score. Synonyms of certain chemical compounds are given in braces {}.

**Supplementary Table S3. List of the targets of active chemical compounds of Jakyak-Gamcho decoction.**

| Herbal medicines | Chemical compounds | Targets |
| --- | --- | --- |
| PlP | (+)-Catechin | ALPL, CA1, CA2, CA3*, CA4, CA5A, CA5B,  CA6, CA7, CA9, CA12, FUT4, PGD, PGF |
| PlP | Palbinone | ARHGEF7, EME1, MMAB |
| PlP | Gallic acid | CA1, CA2, CA3*, CA4, CA5A, CA5B, CA6, CA7,  CA9, CA12, CA14, FUT7, MMP2*, TPMT |
| PlP | Albiflorin | CLIC2, DUSP6, HFE, PDE3B*, PDPK1* |
| PlP | β-Sitosterol | ACBD7, CYP17A1, DHPS, DRAP1, GPBAR1, LTF, MDM2*, MEX3D,  MTOR*, NPC1L1, NR1D2, RAC1*, RANBP2, RUVBL1, TNNC1*, USF1 |
| PlP | Oxypaeoniflorin | ACADS, ALAD*, BLVRA, NUCB1, PRKD2, XRCC6 |
| PlP | Paeoniflorigenone | CLIC2, DRAP1, ECH1, FKBP4, HFE, HOXB1, MEF2A,  NR1D2, NUCB1, RUVBL1, SULT2A1, UNC45A |
| PlP | Paeoniflorin | BLVRA, F13A1, FRS3, HNRNPR, NUCB1, STRBP |
| PlP | Paeonol | MTNR1A*, MTNR1B* |
| PlP/GuF | Kaempferol | ABCB1*, ABCC1, ABCG2*, AHR, AKR1B1, ALOX5*, AR*,  CA12, CA2, CA7, CDK1*, CISD1, CTDSP1, CYP1A2*, CYP1B1,  CYP2D6*, DAPK1, ESRRA, FLT3*, HSD17B1, HSD17B2, MPO,  NOX4, NR1I2, P4HB, PTPRS, SLC2A1*, TYR, UGT3A1, XDH |
| PlP/GuF | Mairin | A1CF, AKR1B10, AKR1B10, AMD1, ARHGEF7, CDCA8,  CPSF4, CYP2R1, DHPS, EIF2AK2, EME1, FOLH1, GPBAR1,  HIGD1B, MDM2*, MEX3D, MMAB, MTOR*, POLB, PTPRN, RAC1*, RNF31, RPS3, S100A8*, SAE1, SAE1, UBA2, UBA2, USF1 |
| PlP/GuF | Sitosterol | CALML3*, CYP17A1, DHPS, DRAP1, GPBAR1, HDLBP,  LTF, NPC1L1, NR1D2, RAC1*, RUVBL1, USF1 |
| GuF | ZINC519174 | CYP19A1*, TAS2R31 |
| GuF | Shinflavanone | ODC1, PTPN1 |
| GuF | Kanzonol B | LTF, ODC1 |
| GuF | Hedysarimcoumestan B | CP1B1 |
| GuF | Enoxolone | AKR1B10, ARHGEF7, CD81, EIF2AK2, EME1, HSD11B1, HSD11B2,  MMAB, PRKCH, PTPN11*, PTPN2*, PTPRJ, RNF31, TGFA |
| GuF | Gancaonin O | AKT1*, CREB1*, PTPRS |
| GuF | 4'-Methoxyglabridin | ACBD7, CALML3*, EIF2AK2, MDM2*,  MEX3D, MTOR*, ODC1, RAC1*, USF1 |
| GuF | Glyuranolide | CITED2, DAPK3, F2R*, HSD11B2, MMAA,  NR1D2, RANBP2, RGS18, RUNX1T1, TJP1 |
| GuF | 3'-Hydroxy-4'-O-methylglabridin | ACBD7, CALML3*, EIF2AK2, MDM2*,  MEX3D, MTOR*, ODC1, RAC1*, USF1 |
| GuF | 3'-Methoxyglabridin | ACBD7, AMD1, CALML3*, EIF2AK2, MDM2*,  MEX3D, MTOR*, ODC1, RAC1*, TGFA, USF1 |
| GuF | Gancaonin M | ALDH2*, CDCA8, RNF31 |
| GuF | 6-Prenylated eriodictyol | ODC1, TAS2R31 |
| GuF | ZINC105741014 | CYP1B1, HSD17B3 |
| GuF | 7-Acetoxy-2-methylisoflavone | ARHGEF7, CPSF4, CYP2R1, EME1, FOLH1, MMAB, PTPRJ |
| GuF | ZINC520945 | ARHGEF7, CPSF4, CYP1A2*, CYP2R1,  EME1, FOLH1, MMAB, PTPRJ |
| GuF | Kanzonol U | ACADVL, ARHGEF7, CPSF4, CYP2R1, EME1, FOLH1 |
| GuF | 8-Prenylated eriodictyol | EME1, ODC1, TAS2R31 |
| GuF | Calycosin | ALDH2*, CYP1B1 |
| GuF | Dehydroglyasperin C | CDCA8 |
| GuF | Liquiritigenin | CYP19A1*, TAS2R31 |
| GuF | Eurycarpin A | ALDH2* |
| GuF | Formononetin | ALDH2*, CYP1B1, IL2* |
| GuF | Gadelaidic acid | CDCA8, DHPS, FAAH*, FABP3, GPR34, GPR174, LPAR1*,  LPAR2*, LPAR3*, LPAR4, LPAR6*, LTF, NR1D2, OXER1, P2RY10, PLA2G4B, PPARA*, PPARD, PPARG*, PTPN7, RNF31 |
| GuF | Gancaonin A | ALDH2* |
| GuF | Gancaonin B | ALDH2* |
| GuF | Gancaonin G | ALDH2*, LTF |
| GuF | Gancaonin H | PTPN1 |
| GuF | Glabranin | ACADVL, ESR1*, ESR2*, HDLBP, ODC1, RNF31, TAS2R31 |
| GuF | Glabrene | TYR |
| GuF | Glabridin | ACBD7, CALML3*, EIF2AK2, HSP90AA1*, MDM2*,  MEX3D, MTOR*, RAC1*, TYR, USF1 |
| GuF | Glabrone | ALDH2*, ODC1, TNNC1* |
| GuF | Glepidotin A | ACADVL, EME1 |
| GuF | Glepidotin B | ACADVL, EME1, REG4 |
| GuF | Glyasperin C | TYR |
| GuF | Glyasperin F | ACADVL, ODC1 |
| GuF | Glyasperin M | ODC1, TNNC1*, USF1 |
| GuF | Glycyrin | TNNC1* |
| GuF | Glycyrol | CDCA8, DHPS, HDLBP |
| GuF | Glycyrrhiza flavonol A | DRAP1 |
| GuF | Glycyrrhizin | GCDH, GRB14, HSD11B2, MS4A1, THRB* |
| GuF | Glypallichalcone | ABCG2*, CYP1B1, MAPT*, TNFRSF1A*, TUBB1 |
| GuF | Glyzaglabrin | ALDH2* |
| GuF | Isoformononetin | ALDH2*, IL2* |
| GuF | 5E-Eicosenoic acid | FAAH*, LPAR3*, LPAR4, NR1D2, OXER1, PLA2G4B, RANBP2, SLCO2A1 |
| GuF | Inflacoumarin A | ALDH2*, S100A8*, HSD17B3, PTPRN, TGFA |
| GuF | Isoglycyrol | ACBD7, CALML3*, CDCA8, EIF2AK2, MDM2*,  MEX3D, MTOR*, NR1D2, RAC1*, TGFA, USF1 |
| GuF | Isolicoflavonol | CYP19A1*, XDH |
| GuF | Isoliquiritigenin | AKR1B1, CYP1A2*, CYP3A4* |
| GuF | Isoliquiritin | AKR1B1, AKR1B1, SLC28A3, SLC5A2, TYR |
| GuF | Isorhamnetin | CA2, CA4, CA7, CA12, CYP1A1*,  CYP1B1, CYP1B1, P4HB, XDH |
| GuF | Isotrifoliol | CYP1B1, HSD17B3 |
| GuF | Jaranol | ALDH2*, CREB1*, CYP1B1, P4HB |
| GuF | Kanzonol W | CDCA8 |
| GuF | Licoagroisoflavone | ACBD7, ALDH2*, CDCA8, DHPS,  MDM2*, MEX3D, MTOR*, TGFA |
| GuF | Licochalcone A | ABCG2*, LTF, TNFRSF1A* |
| GuF | Licochalcone B | ABCG2*, TNFRSF1A* |
| GuF | Licochalcone G | LTF |
| GuF | Licoisoflavanone | CDCA8, ODC1 |
| GuF | Licoricone | ALDH2* |
| GuF | Liquiritin | TAS2R31, TYR |
| GuF | Lupiwighteone | ALDH2, CDCA8, RNF31 |
| GuF | Naringenin | ABCC1, CA4, CA7, CA12, CBR1, CYP1B1, HSD17B1  CYP1A2*, CYP19A1*, CYP3A4*, SHBG, TAS2R31 |
| GuF | Odoratin | ALDH2*, CYP1B1, PPARG* |
| GuF | Ononin | ALDH2*, IL2*, SLC5A2 |
| GuF | Phaseol | AMD1, CALML3*, CDCA8, DHPS, HSD17B3, RNF31 |
| GuF | Phaseolinisoflavan | CDCA8, USF1 |
| GuF | Pinocembrin | CYP19A1*, CYP1B1, TAS2R31 |
| GuF | Quercetin | ABCB1*, ABCC1, ABCG2*, AKR1B1, AKT1*, ALK*, ALOX5*, ALOX12*,  ALOX15*, ATP5B, AURKB, AVPR2*, AXL, BACE1, CA1, CA2, CA3*, CA4, CA5A, CA6, CA7, CA9, CA12, CA14, CAMK2B*, CCR4*, CDK1*, CSNK2A1, CXCR1*, CYP1A1*, CYP1A2*, CYP1B1, CYP2C8*, CYP2C9*, DAPK1, DRD4*, EGFR*, ELAVL1, F2*, FLT3*, GLO1, GPR35*, GSK3B*, HCK*, HIBCH, HSD17B2, IGF1R*, KDR*, MAOA*, MCL1*, MET*, MMP2*, MMP3*, MMP9*, MMP13*, MPO, NEK2, NEK6, NOX4, NUAK1, P4HB, PIK3R1*, PIM1, PKN1, PLA2G1B, PTK2*, PTPRS, PYGL, SLC2A2, STK17B, XDH |
| GuF | Quercetin 3,3'-dimethyl ether | AKR1B1, CYP1B1, P4HB |
| GuF | Semilicoisoflavone B | TNNC1* |
| GuF | Shinpterocarpin | ACBD7, AMD1, CALML3*, CDCA8, DHPS, EIF2AK2, MDM2*,  MEX3D, MTOR*, ODC1, PTPRN, RAC1*, RPS3, S100A8*, TGFA |
| GuF | Sigmoidin B | TAS2R31 |
| GuF | Xambioona | HDLBP, ODC1, TNNC1* |

PlP, *Paeonia lactiflora* Pallas; GuF, *Glycyrrhiza uralensis* Fischer; *, pain-associated targets.

**Supplementary Table S4. Docking scores of the active chemical compounds of Jakyak-Gamcho decoction with the hub targets.**

| Chemical compounds | Hub targets | | | | |
| --- | --- | --- | --- | --- | --- |
|  | AKT1 | EGFR | HSP90AA1 | LPAR1 | PIK3R1 |
| (+)-Catechin | -5.9 | -7.7 | -7.6 | -5.7 | -6.1 |
| 3'-Hydroxy-4'-O-methylglabridin | -5.6 | -9.0 | -7.8 | -6.1 | -6.8 |
| 3'-Methoxyglabridin | -5.3 | -8.8 | -8.0 | -5.7 | -6.8 |
| 4'-Methoxyglabridin | -5.6 | -9.1 | -7.8 | -6.2 | -6.4 |
| 5E-Eicosenoic acid | -3.8 | -6.5 | -5.2 | -4.3 | -3.9 |
| Albiflorin | -5.9 | -8.5 | -8.0 | -5.9 | -6.4 |
| Calycosin | -7.5 | -9.1 | -8.3 | -7.0 | -7.9 |
| Enoxolone | -5.4 | -9.3 | -7.4 | -7.2 | -7.1 |
| Eurycarpin A | -6.3 | -9.2 | -7.6 | -6.1 | -6.4 |
| Formononetin | -5.5 | -7.9 | -7.2 | -6.0 | -5.7 |
| Gadelaidic acid | -3.6 | -6.3 | -5.6 | -4.6 | -4.1 |
| Gallic acid | -5.3 | -6.4 | -5.6 | -4.4 | -4.9 |
| Gancaonin A | -5.5 | -8.7 | -7.5 | -6.3 | -6.0 |
| Gancaonin B | -5.7 | -8.9 | -7.2 | -6.2 | -6.1 |
| Gancaonin G | -5.9 | -8.3 | -8.1 | -6.2 | -6.3 |
| Gancaonin M | -5.3 | -8.7 | -8.1 | -6.2 | -6.3 |
| Gancaonin O | -5.7 | -9.0 | -8.1 | -6.6 | -6.4 |
| Glabranin | -5.4 | -8.3 | -8.3 | -6.7 | -6.6 |
| Glabridin | -5.6 | -8.9 | -8.3 | -5.9 | -6.8 |
| Glabrone | -6.5 | -8.9 | -7.6 | -6.6 | -6.7 |
| Glyasperin M | -6.6 | -9.0 | -7.6 | -7.1 | -6.4 |
| Glycyrin | -5.5 | -8.8 | -7.5 | -6.1 | -5.8 |
| Glycyrrhizin | -4.7 | -7.9 | -6.3 | -6.4 | -3.6 |
| Glypallichalcone | -6.2 | -7.9 | -7.1 | -5.7 | -5.5 |
| Glyuranolide | -5.9 | -9.2 | -7.3 | -6.9 | -7.3 |
| Glyzaglabrin | -6.4 | -8.5 | -7.3 | -6.2 | -6.7 |
| Inflacoumarin A | -5.9 | -8.5 | -7.8 | -7.3 | -6.6 |
| Isobavachin | -6.3 | -8.2 | -8.2 | -6.7 | -6.8 |
| Isoformononetin | -6.1 | -7.8 | -7.1 | -5.8 | -5.9 |
| Isoglycyrol | -5.8 | -9.9 | -9.1 | -7.4 | -6.9 |
| Isolicoflavonol | -6.1 | -9.4 | -8.3 | -6.4 | -6.5 |
| Isoliquiritigenin | -6.5 | -7.9 | -7.0 | -6.0 | -6.1 |
| Isorhamnetin | -6.0 | -8.1 | -7.7 | -5.9 | -6.3 |
| Jaranol | -5.6 | -7.8 | -7.1 | -5.7 | -6.0 |
| Kaempferol | -6.3 | -7.8 | -7.4 | -5.8 | -6.1 |
| Licoagroisoflavone | -5.6 | -9.6 | -8.5 | -6.1 | -7.0 |
| Licochalcone A | -5.4 | -8.2 | -7.4 | -6.5 | -6.0 |
| Licochalcone B | -5.9 | -7.7 | -7.5 | -5.6 | -5.8 |
| Licoricone | -5.5 | -8.7 | -7.4 | -6.0 | -6.4 |
| Liquiritigenin | -6.0 | -7.9 | -7.4 | -6.2 | -6.1 |
| Lupiwighteone | -5.6 | -8.8 | -8.0 | -6.2 | -6.2 |
| Mairin | -5.0 | -9.8 | -7.2 | -8.4 | -6.8 |
| Naringenin | -5.9 | -7.8 | -7.5 | -6.0 | -6.1 |
| Odoratin | -5.3 | -8.1 | -7.1 | -5.8 | -6.0 |
| Ononin | -6.2 | -9.1 | -8.1 | -5.9 | -6.5 |
| Oxypaeoniflorin | -6.1 | -8.6 | -8.4 | -5.9 | -6.6 |
| Paeonol | -5.3 | -5.7 | -5.5 | -4.4 | -4.5 |
| Phaseol | -5.8 | -9.4 | -8.3 | -6.6 | -6.5 |
| Pinocembrin | -5.5 | -7.7 | -7.8 | -6.2 | -6.0 |
| Quercetin | -6.4 | -8.0 | -7.6 | -5.9 | -6.3 |
| Semilicoisoflavone B | -5.9 | -9.4 | -8.2 | -6.1 | -6.8 |
| Shinpterocarpin | -6.3 | -10.0 | -8.0 | -6.5 | -6.6 |
| Sitosterol | -4.5 | -8.8 | -8.3 | -6.1 | -5.8 |
| Xambioona | -5.9 | -10.5 | -9.1 | -8.0 | -7.7 |
| ZINC519174 | -6.2 | -7.9 | -7.4 | -6.2 | -6.1 |
| ZINC520945 | -5.3 | -7.8 | -7.2 | -5.8 | -5.7 |
| β-Sitosterol | -4.4 | -8.8 | -8.3 | -6.1 | -5.9 |
